# Oral preventive medications for migraine in adults aged 18–65: a network meta-analysis

**DOI:** 10.3389/fphar.2025.1620887

**Published:** 2025-09-04

**Authors:** Jianping Wu, Jun Wu, Jian Zhang, Bingbing Liu, Xinyuan Song, Yuanjie Wu, Fanian Tian, Yanbing Ding, Ping Wang

**Affiliations:** ^1^ Hubei University of Chinese Medicine, Wuhan, Hubei, China; ^2^ Engineering Research Center of TCM Protection Technology and New Product Development for the Elderly Brain Health, Ministry of Education, Wuhan, Hubei, China; ^3^ Hubei Provincial Hospital of Traditional Chinese Medicine, Wuhan, Hubei, China; ^4^ Hubei Key Laboratory of Theory and Application Research of Liver and Kidney in Traditional Chinese Medicine, Affiliated Hospital of Hubei University of Chinese Medicine, Wuhan, Hubei, China; ^5^ Hubei Shizhen Laboratory, Wuhan, Hubei, China; ^6^ Hubei Province Academy of Traditional Chinese Medicine, Wuhan, Hubei, China; ^7^ Department of Otolaryngology, People’s Hospital of Xinzhou, Wuhan, Hubei, China

**Keywords:** network meta-analysis, adults, migraine, oral medications, preventive treatment

## Abstract

**Background:**

Migraine is a highly prevalent neurological disorder that significantly impairs quality of life. Understanding the comparative effectiveness and safety of oral preventive medications is essential to guide treatment decisions in adult patients. This study aims to evaluate and compare the efficacy and safety of oral pharmacological therapies for migraine prevention in adults using Network Meta-Analysis.

**Methods:**

A comprehensive search was conducted across The Cochrane Library, PubMed, SCOPUS, and Embase databases until 15 December 2024 to find relevant studies on preventing migraine among adult populations. Clinical trials involving adult individuals with migraine who received oral pharmacological interventions were included. Per the Preferred Reporting Items for Systematic Reviews and Meta-Analyses (PRISMA) guidelines, data extraction was independently conducted by five researchers in duplicate. Model choice was based on heterogeneity with random-effects used for I^2^ ≥ 50% and fixed-effects for I^2^ < 50%. The main endpoint was the monthly frequency of migraine attacks. Secondary endpoints encompassed the response rate of ≥50%, migraine duration, pain intensity, and quality of life (QoL). Adverse events were assessed.

**Results:**

From the 17,443 identified citations, we included 44 trials (4,612 participants) in our analysis. Topiramate, valproate, and propranolol demonstrated significant efficacy in the prevention of migraines. Memantine, melatonin, and vitamin D3 also showed potential preventive effects. Combination therapies, such as flunarizine plus topiramate, valproate plus magnesium, or folic plus pyridoxine, were associated with greater efficacy in migraine prevention compared to monotherapy and with a lower incidence of adverse events. Topiramate, flunarizine, propranolol, valproate, amitriptyline, cinnarizine, and nortriptyline were associated with improvements in quality of life (QoL), but these findings were based on limited evidence. Valsartan and a-dihydroergocryptine were linked to reduced migraine frequency, but these results were largely derived from single studies and require confirmation through larger, high-quality trials.

**Conclusion:**

This network meta-analysis confirmed the significant efficacy of topiramate, valproate, and propranolol in migraine prevention and identified potential benefits of memantine, melatonin, vitamin D3, and combination therapies. These findings provide evidence-based treatment options for migraine prevention and suggest promising directions for future research.

**Systematic Review Registration:**

https://www.crd.york.ac.uk/PROSPERO/display_record.php?ID=CRD42024621316, Identifier: PROSPERO, CRD42024621316.

## Introduction

Migraine, a chronic neurological disorder, causes severe headaches and can potentially lead to disability. Symptoms like nausea and sensitivity to light and sound can last up to 72 h (Ashina, 2020). Migraine is associated with psychiatric disorders, atopic diseases, and cardiovascular disease, among others, with mutual influences among these conditions (Terhart et al., 2025). Migraine affects over one billion people worldwide (GBD, 2016 Headache Collaborators, 2018), predominantly adults (Leonardi et al., 2020). The high disability rate associated with migraine significantly impairs patients’ quality of life (QoL) and productivity, creating significant financial strain on individuals and the community (GBD, 2016 Headache Collaborators, 2018; Ashina et al., 2021).

The 2018 International Classification of Headache Disorders, Third Edition (ICHD-III) (Arnold, 2018) provides standardized diagnostic criteria, thereby enhancing the accuracy of migraine diagnosis and aiding in its ongoing management and treatment. Around 40% of individuals with episodic migraine may benefit from preventive therapy to reduce migraine frequency and delay progression, yet only 17% receive it (Silberstein et al., 2025; L et al., 2007). Migraine treatment is categorized into acute and preventive treatments, with a preventive treatment assessment recommended after each acute episode (Kumar and Kadian, 2025). Medications for migraine prophylaxis mainly include anticonvulsants, β-blockers, angiotensin receptor blockers/ACE inhibitors, calcium antagonists, serotonin antagonists, antidepressants, CGRP monoclonal antibodies (mAbs), onabotulinumtoxin A, and gepants (Puledda et al., 2024a). Additionally, coenzyme Q10 (CoQ10), riboflavin, omega-3 fatty acids, melatonin, and vitamin D3 have shown potential in migraine prevention (Tseng et al., 2024; Puliappadamb et al., 2022; Sazali et al., 2021; Okoli et al., 2019). Agents such as memantine may also exert beneficial effects through modulation of glutamate signaling (Charles, 2021). CGRP drugs, typically administered by monthly or quarterly injection, may improve patient adherence due to their lower dosing frequency (Varnado et al., 2022; Sacco et al., 2022). However, injections can be inconvenient, requiring professional administration and potentially causing injection site reactions (Mechtler et al., 2022; Suzuki et al., 2025). Moreover, these drugs are unavailable in most countries globally (Kiarashi et al., 2021; Tana et al., 2024; Puledda et al., 2024b), highlighting the unique advantages of oral preventive treatments.

Although prophylactic treatments for migraine have demonstrated effectiveness and safety, most evidence focuses on individual drugs. There is a lack of direct comparisons between different medications (Versijpt et al., 2024; Raffaelli et al., 2023; Deligianni et al., 2023). This limitation in evidence makes it difficult for clinical guidelines to provide a clear hierarchical structure for the selection of prophylactic medications (Arnold, 2018). Specifically, most existing studies fail to directly compare the efficacy and safety of different prophylactic drugs, which poses challenges for clinicians in selecting appropriate treatments. Therefore, more research is needed to fill this knowledge gap and offer more specific guidance for clinical practice.

Since adults are primarily affected by migraine (Leonardi et al., 2020), this study evaluated the efficacy and safety of oral prophylactic drugs in migraine patients aged 18–65 years using a network meta-analysis (NMA). The upper age limit was set at 65 because patients aged 65 and above are generally considered elderly, a group that typically has multiple comorbidities and different drug metabolism and tolerability compared with younger patients (Jhaveri et al., 2014; Ngcobo, 2025). Therefore, to ensure the accuracy and comparability of the study results, this study focused on adults aged 18–65. Future studies should consider a broader age range to better address the needs of different patients.

This network meta-analysis aimed to compare the efficacy, safety, and tolerability of oral preventive drugs in adults aged 18–65 years with migraine, providing evidence-based guidance for clinical treatment selection.

## Methods

This research report, including randomized clinical trials (RCTs), was registered in the International Prospective Register of Systematic Reviews (PROSPERO, CRD42024621316, 13 December 2024) and adhered to the principles of the Preferred Reporting Items for Systematic Reviews and Meta-Analyses (PRISMA) guidelines (Moher et al., 2009; Liberati et al., 2009; Page et al., 2021).

### Search protocol and study inclusion

The Cochrane Library, PubMed, Scopus, and Embase databases were searched for eligible RCTs published from database inception until 15 December 2024 (eAppendix Supplement 1). Next, four researchers (J.P.W., J.Z., J.W., and Y.J.W.) independently screened the titles and abstracts in duplicate. Four researchers (J.P.W., B.B.L., J.W., and F.N.T.) conducted two rounds of full-text screening of the selected studies based on the PICOT principles.

Study Population: Individuals aged 18–65 years with a confirmed diagnosis of migraine according to the ICHD-III (L et al., 2007), or highly consistent with the classification criteria of the International Headache Society (IHS).
Interventions: Oral drugs for migraine prevention.
Comparisons: Two oral preventive drugs were directly compared with each other or with a placebo.
Outcomes: Monthly frequency of migraine attacks, ≥50% responder rates (defined as a 50% decline in migraine frequency), migraine duration (hours), pain intensity (subjective pain score), and QoL (evaluated using the migraine disability assessment questionnaire, MIDAS) were measured. Disagreements were resolved through discussions, with a third party (P.W.) arbitrating when needed.

### Data extraction

The included studies were allocated by five researchers (J.P.W., X.Y.S., Y.B.D., J.W., and Y.J.W.) and their characteristics and outcomes were independently extracted into an Excel spreadsheet in duplicate. For research involving repeated assessments, the last assessment was chosen for analysis. Discrepancies were resolved through discussion.

### Outcomes

The main endpoint was the monthly frequency of migraine attacks post-treatment.

The secondary endpoints included ≥50% response rate, migraine duration, pain intensity, and QoL, as defined above. Additionally, data on adverse events were extracted to evaluate treatment safety.

### Quality evaluation and risk of bias

The caliber of the filtered studies was evaluated using the Cochrane Risk of Bias Tool, version 2 (Higgins et al., 2019). Every study was independently evaluated by two researchers (J.P.W and J.W).

### Statistical analysis

The primary and secondary endpoints were evaluated using frequentist network meta-analyses grounded in graph-theoretical methodology employing the R package Netmeta (version 3.1.1). The effect size was quantified using the risk ratio (RR) and its corresponding 95% confidence interval (CI) for binary outcomes and the ratio of the mean (ROM) to its corresponding 95% CI for continuous variables. In all evaluations, the control group was the placebo.

Therapeutic efficacy and ranking for each outcome were determined using P-score, consistent with surface under the cumulative ranking curve (SUCRA) results. Heterogeneity in the intra- and inter-study comparisons was quantified using Q statistics and the I^2^ statistic. Transitivity was evaluated by examining key study characteristics and analyzing local consistency via node splitting analysis of direct and indirect evidence and overall consistency via design-by-treatment interaction analysis.

To assess the impact of baseline study confounders on the primary endpoint, a secondary random-effects meta-regression was performed using the metafor 4.8.0 R package. Covariates included patient age, the proportion of males, follow-up duration, diagnostic method, and migraine with aura. These covariates were selected in accordance with the IHS guidelines and relevant U.S. guidelines (Arnold, 2018; Silberstein et al., 2025). Covariates were considered non-significant if P > 0.05 and the 95% CI included the null value. The primary data analysis was conducted from January 2025 to February 2025.

## Results

Among 17,443 citations retrieved, the titles and abstracts of 11,350 citations were screened after removing duplicates. After screening the full texts of 376 articles, the systematic review and NMA included 44 clinical trial articles (4,612 participants) (Carrieri et al., 1988; Steiner et al., 1988; Stewart et al., 1988; Tuca et al., 1989; Gawel et al., 1992; Peikert et al., 1996; Schoenen et al., 1999; Lucking et al., 1988; Bussone et al., 1999; Diener et al., 2001a; Diener et al., 2001b; Pradalier et al., 2001; Edwards et al., 2003; Silvestrini et al., 2003; Mei et al., 2004; Sandor et al., 2005; Silberstein et al., 2006; Ashtari et al., 2008; Afshari et al., 2011; Bostani et al., 2013; Sadeghi et al., 2015; Assarzadegan et al., 2016; Noruzzadeh et al., 2016; Choudhary et al., 2017; Hesami et al., 2018a; Hesami et al., 2018b; Gazerani et al., 2019; Hajihashemi et al., 2019; Shanmugam et al., 2019; Ghorbani et al., 2020; Chowdhury et al., 2022; Hedayat et al., 2022; Sherafat et al., 2022; Balali et al., 2024; Mehramiri et al., 2024; Mosarrezaii et al., 2025; Roghani et al., 2024; Diener et al., 1996; Keskinbora and Aydinli, 2008; Luo et al., 2012; Gonçalves et al., 2016; Askari et al., 2017; Ebrahimi-Monfared et al., 2017; Khani et al., 2021) (Supplementary Figure S1). Among these, 37 studies (Carrieri et al., 1988; Steiner et al., 1988; Stewart et al., 1988; Tuca et al., 1989; Gawel et al., 1992; Peikert et al., 1996; Schoenen et al., 1999; Lucking et al., 1988; Bussone et al., 1999; Diener et al., 2001a; Diener et al., 2001b; Pradalier et al., 2001; Edwards et al., 2003; Silvestrini et al., 2003; Mei et al., 2004; Sandor et al., 2005; Silberstein et al., 2006; Ashtari et al., 2008; Afshari et al., 2011; Bostani et al., 2013; Sadeghi et al., 2015; Assarzadegan et al., 2016; Noruzzadeh et al., 2016; Choudhary et al., 2017; Hesami et al., 2018a; Hesami et al., 2018b; Gazerani et al., 2019; Hajihashemi et al., 2019; Shanmugam et al., 2019; Ghorbani et al., 2020; Chowdhury et al., 2022; Hedayat et al., 2022; Sherafat et al., 2022; Balali et al., 2024; Mehramiri et al., 2024; Mosarrezaii et al., 2025; Roghani et al., 2024) and 7 studies (Diener et al., 1996; Keskinbora and Aydinli, 2008; Luo et al., 2012; Gonçalves et al., 2016; Askari et al., 2017; Ebrahimi-Monfared et al., 2017; Khani et al., 2021) were two-arm and three-arm RCTs, respectively. The features of the included studies are detailed in Supplementary Table S1 18 studies (1,602 patients) (Ashtari et al., 2008; Afshari et al., 2011; Bostani et al., 2013; Sadeghi et al., 2015; Assarzadegan et al., 2016; Noruzzadeh et al., 2016; Hesami et al., 2018a; Hajihashemi et al., 2019; Ghorbani et al., 2020; Hedayat et al., 2022; Sherafat et al., 2022; Balali et al., 2024; Mehramiri et al., 2024; Mosarrezaii et al., 2025; Roghani et al., 2024; Askari et al., 2017; Ebrahimi-Monfared et al., 2017; Khani et al., 2021) were conducted in Iran, constituting approximately 34.7% of the total participants. The bias risk assessment indicated that, among the included studies, 59% were rated as having a medium risk of bias, and 41% were rated as having a low risk of bias (Supplementary Table S1). Supplementary Figure S2, S3 illustrate the factors influencing the overall risk of bias score (Mcguinness and Higgins, 2021).

### Primary outcome

#### Migraine frequency

Migraine frequency data were reported in 30 of the 44 included studies (n = 3,162) (Carrieri et al., 1988; Steiner et al., 1988; Stewart et al., 1988; Lucking et al., 1988; Bussone et al., 1999; Diener et al., 2001a; Diener et al., 2001b; Pradalier et al., 2001; Silvestrini et al., 2003; Ashtari et al., 2008; Afshari et al., 2011; Bostani et al., 2013; Sadeghi et al., 2015; Assarzadegan et al., 2016; Noruzzadeh et al., 2016; Choudhary et al., 2017; Hesami et al., 2018a; Gazerani et al., 2019; Hajihashemi et al., 2019; Shanmugam et al., 2019; Ghorbani et al., 2020; Hedayat et al., 2022; Balali et al., 2024; Mosarrezaii et al., 2025; Roghani et al., 2024; Keskinbora and Aydinli, 2008; Luo et al., 2012; Askari et al., 2017; Ebrahimi-Monfared et al., 2017; Khani et al., 2021). Given low heterogeneity (I^2^ = 0%, Q = 3.84, P = 0.70), a fixed-effects model was used.

**FIGURE 1:**
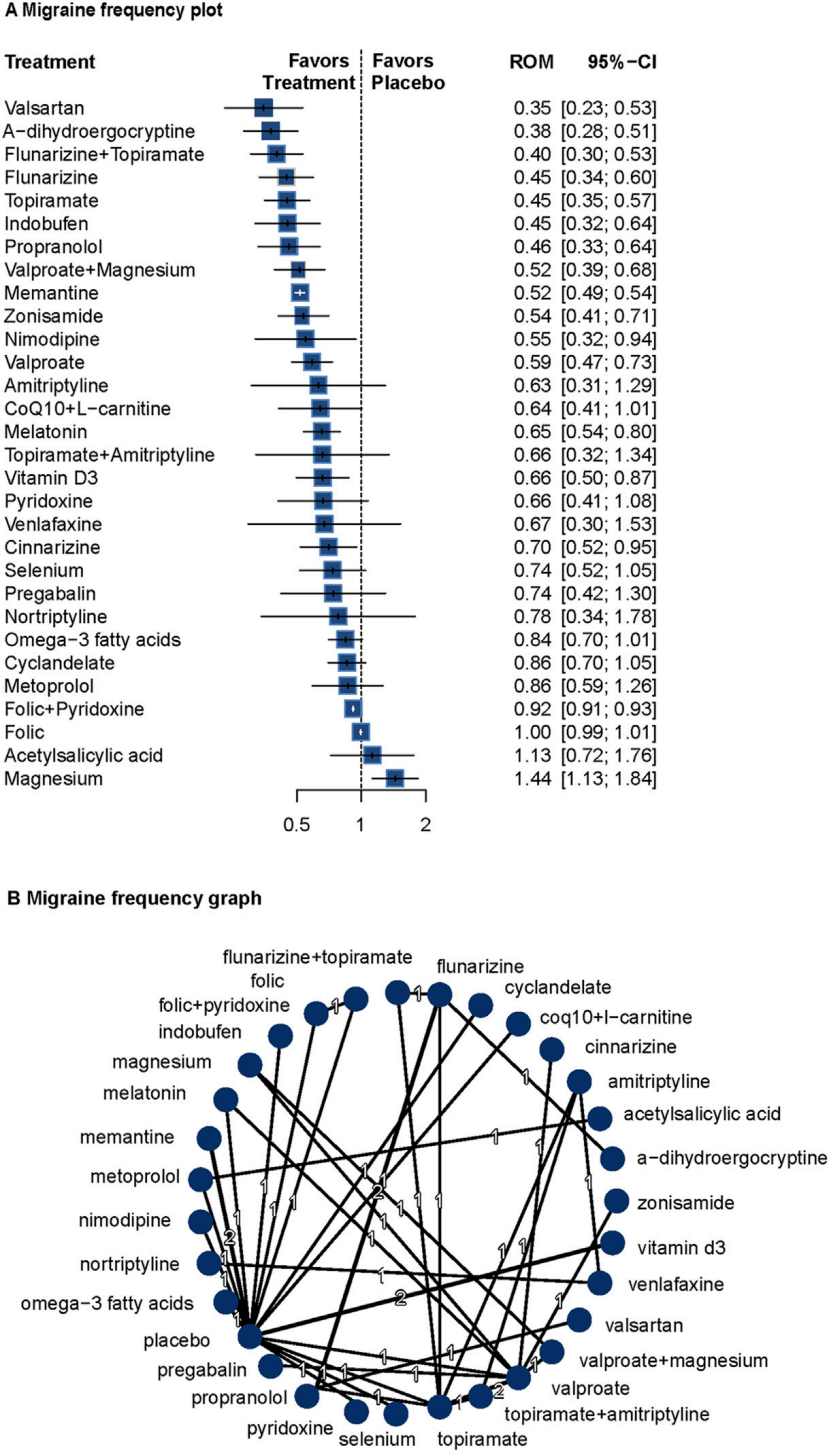
Association of treatment with migraine frequency. **(A)** Migraine frequency plot. **(B)** Migraine frequency graph. Forest plot **(A)** and network graph **(B)** displaying the comparative efficacy of various treatments in reducing the frequency of migraine attacks in adult patients. The data includes the ratio of means (RoM) for each treatment compared with placebo, calculated using a fixed-effects model due to heterogeneity (I^2^ = 0%). Significant reductions were observed with valsartan, flunarizine with topiramate, flunarizine, topiramate, indobufen, propranolol, volproate with magnesium, memantine, zonisamide, nimodipine, valproate, CoQ10 with l-carnitine, melatonin, vitamin D3, cinnarizine, and folic with pyridoxine. The analysis was based on 30 studies (Carrieri et al., 1988; Steiner et al., 1988; Stewart et al., 1988; Lucking et al., 1988; Bussone et al., 1999; Diener et al., 2001a; Diener et al., 2001b; Pradalier et al., 2001; Silvestrini et al., 2003; Ashtari et al., 2008; Afshari et al., 2011; Bostani et al., 2013; Sadeghi et al., 2015; Assarzadegan et al., 2016; Noruzzadeh et al., 2016; Choudhary et al., 2017; Hesami et al., 2018a; Gazerani et al., 2019; Hajihashemi et al., 2019; Shanmugam et al., 2019; Ghorbani et al., 2020; Hedayat et al., 2022; Balali et al., 2024; Mosarrezaii et al., 2025; Roghani et al., 2024; Keskinbora and Aydinli, 2008; Luo et al., 2012; Askari et al., 2017; Ebrahimi-Monfared et al., 2017; Khani et al., 2021) with 3162 patients. CoQ10 indicates Coenzyme Q10, and folic indicates folic acid.

Compared with placebo, valsartan (ROM = 0.35; 95%CI = 0.23–0.53), α-dihydroergocryptine (ROM = 0.38; 95%CI = 0.28–0.51), flunarizine plus topiramate (ROM = 0.40; 95%CI = 0.30–0.53), flunarizine (ROM = 0.45; 95%CI = 0.34–0.60), and topiramate (ROM = 0.45; 95%CI = 0.35–0.57) were most effective in reducing migraine frequency, showing both statistical and clinical significance. Several other agents also demonstrated significant effects. The network diagram and forest plots are shown in Figure 1. According to the P-score, the top three treatments were valsartan, a-dihydroergocryptine, and flunarizine plus topiramate. For detailed results and additional information on the comparative effectiveness of all included treatments, please refer to Supplementary Table S2. The funnel plots and Egger’s test revealed no significant publication bias (Supplementary Figure S4). Supplementary Table S3 presents the results for pairwise comparisons for all interventions (league table), and Supplementary Figure S5 shows the details for the network inconsistency test. The heat map for frequency is presented in Supplementary Figure S6.

Covariate meta-regression analyses indicated that age, follow-up duration, migraine with aura, diagnostic criteria, and proportion of males did not significantly influence the results (Supplementary Table S4).

These findings confirm the efficacy of topiramate, valproate, and propranolol, and suggest potential roles for memantine, melatonin, and vitamin D3. Combination therapies such as flunarizine plus topiramate and valproate plus magnesium also showed significant benefits.

### Secondary outcomes

#### Responder rates ≥50%

**FIGURE 2:**
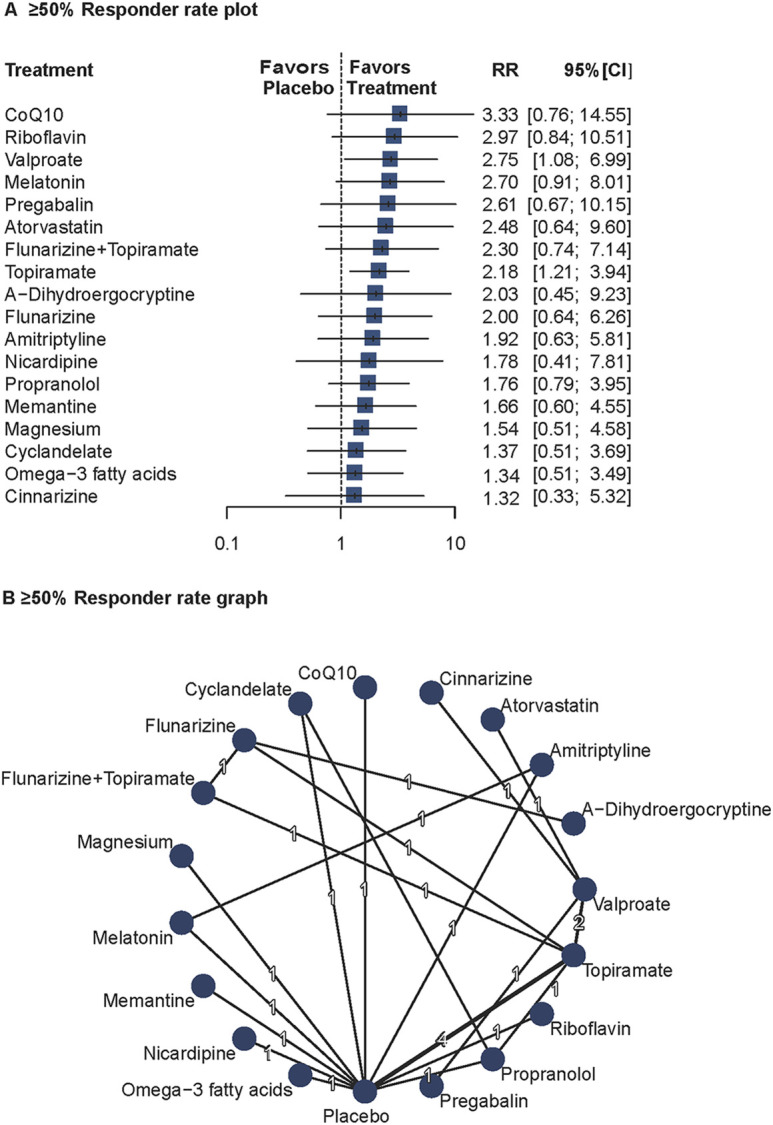
Association of Treatment With 50% or Greater Responder Rate. **(A)** ≥ 50% Responder rate plot. **(B)** ≥ 50% Responder rate graph. Forest plot **(A)** and network graph **(B)** displaying treatments achieving at least a 50% reduction in headache frequency, defined as the number of patients with at least 50% headache frequency reduction after treatment compared with baseline. Risk ratios (RRs) were calculated using a random effects model due to significant heterogeneity (I^2^ = 72.7%). Valproate, and topiramate showed significant efficacy compared with placebo. Data were derived from 20 studies (Tuca et al., 1989; Peikert et al., 1996; Schoenen et al., 1999; Bussone et al., 1999; Pradalier et al., 2001; Edwards et al., 2003; Silvestrini et al., 2003; Mei et al., 2004; Sandor et al., 2005; Silberstein et al., 2006; Afshari et al., 2011; Bostani et al., 2013; Choudhary et al., 2017; Hesami et al., 2018a; Hesami et al., 2018b; Shanmugam et al., 2019; Chowdhury et al., 2022; Diener et al., 1996; Luo et al., 2012; Gonçalves et al., 2016) involving 1994 patients.

Twenty studies (n = 1994) reported ≥50% responder rates (Tuca et al., 1989; Peikert et al., 1996; Schoenen et al., 1999; Bussone et al., 1999; Pradalier et al., 2001; Edwards et al., 2003; Silvestrini et al., 2003; Mei et al., 2004; Sandor et al., 2005; Silberstein et al., 2006; Afshari et al., 2011; Bostani et al., 2013; Choudhary et al., 2017; Hesami et al., 2018a; Hesami et al., 2018b; Shanmugam et al., 2019; Chowdhury et al., 2022; Diener et al., 1996; Luo et al., 2012; Gonçalves et al., 2016). A random-effects model was used due to significant heterogeneity (I^2^ = 72.7%, Q = 17.96, P = 0.003). Valproate (RR = 2.75; 95%CI = 1.08–6.99) and topiramate (RR = 2.18; 95%CI = 1.21–3.94) showed significantly higher responder rates than placebo. The network diagram and forest plots are shown in Figure 2. Full results, including P-score rankings, publication bias assessments, and network inconsistency tests, are detailed in Supplement 1. Pairwise comparisons are summarized in Supplementary Table S5. The heat map for ≥50% responder rates is presented in Supplementary Figure S9.

According to the results of the Q-statistic heterogeneity analysis, further sensitivity analysis was conducted on the follow-up duration. After excluding four studies with follow-up <16 weeks (Silberstein et al., 2006; Afshari et al., 2011; Bostani et al., 2013; Choudhary et al., 2017), heterogeneity was significantly reduced (I^2^ = 50.7%, Q = 6.09, P = 0.107).

These findings confirm the efficacy of valproate and topiramate in migraine prophylaxis and suggest that study duration may affect the consistency of responder rate outcomes.

#### Pain intensity

**FIGURE 3:**
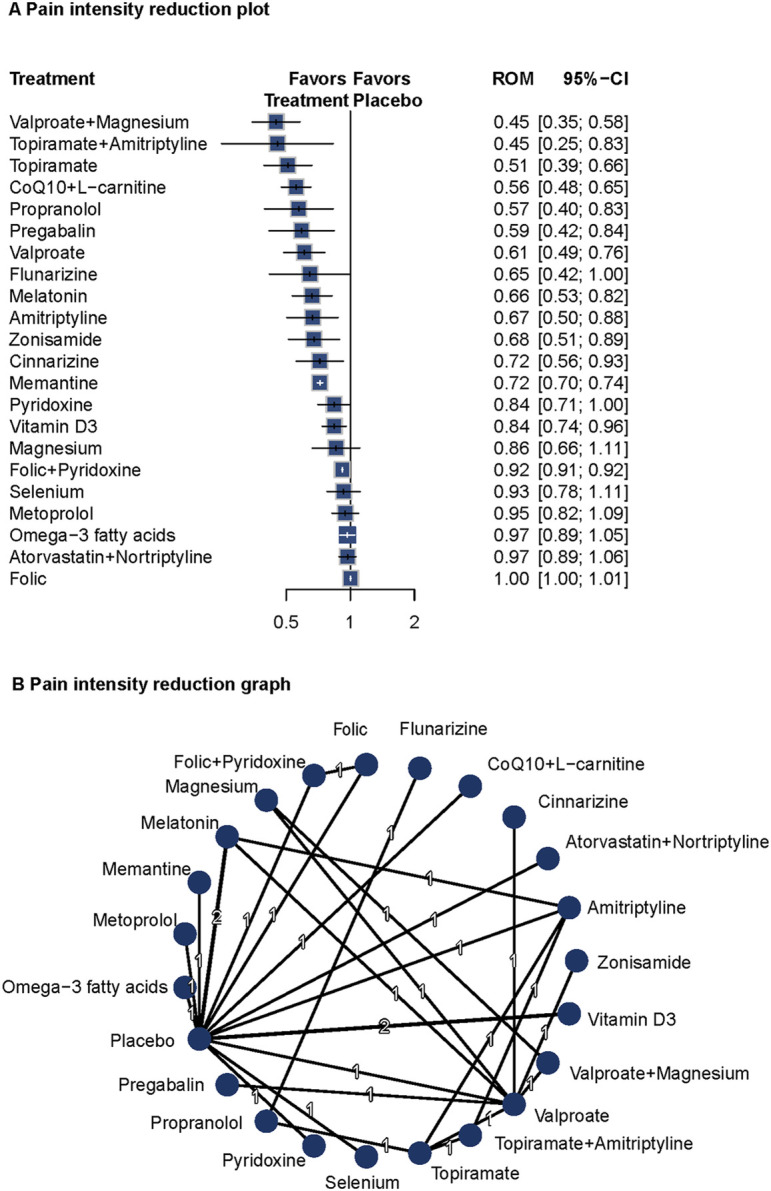
Association of Treatment With Pain Intensity. **(A)** Pain intensity reduction plot. **(B)** Pain intensity reduction graph. Forest plot **(A)** and network graph **(B)** displaying treatments that were associated with a significant reduction of the self-reported intensity of headaches. Ratio of means (RoM) values were calculated using a fixed-effects model (I^2^ = 17.8%). Effective treatments included propranolol with valproate with magnesium, topiramate with amitriptyline, topiramate, CoQ10 with L-carnitine, propranolol, pregabalin, valproate, melatonin, amitriptyline, zonisamide, cinnarizine, memantine, and vitamin D3. The analysis incorporated 20 studies (Steiner et al., 1988; Gawel et al., 1992; Pradalier et al., 2001; Ashtari et al., 2008; Afshari et al., 2011; Bostani et al., 2013; Sadeghi et al., 2015; Assarzadegan et al., 2016; Noruzzadeh et al., 2016; Hesami et al., 2018a; Gazerani et al., 2019; Hajihashemi et al., 2019; Ghorbani et al., 2020; Sherafat et al., 2022; Balali et al., 2024; Keskinbora and Aydinli, 2008; Gonçalves et al., 2016; Askari et al., 2017; Ebrahimi-Monfared et al., 2017; Khani et al., 2021).

Twenty studies (n = 1809) reported pain intensity data (Steiner et al., 1988; Gawel et al., 1992; Ashtari et al., 2008; Afshari et al., 2011; Bostani et al., 2013; Sadeghi et al., 2015; Assarzadegan et al., 2016; Noruzzadeh et al., 2016; Hesami et al., 2018a; Gazerani et al., 2019; Hajihashemi et al., 2019; Ghorbani et al., 2020; Sherafat et al., 2022; Balali et al., 2024; Keskinbora and Aydinli, 2008; Gonçalves et al., 2016; Askari et al., 2017; Ebrahimi-Monfared et al., 2017; Khani et al., 2021). A fixed-effects model was used due to low heterogeneity (I^2^ = 17.8%, Q = 3.65, P = 0.30). Compared with placebo, valproate plus magnesium (ROM = 0.45; 95%CI = 0.35–0.58), topiramate plus amitriptyline (ROM = 0.45; 95%CI = 0.25–0.83), topiramate (ROM = 0.51; 95%CI = 0.39–0.66), CoQ10 plus L-carnitine (ROM = 0.51; 95%CI = 0.39–0.66), propranolol (ROM = 0.57; 95%CI = 0.40–0.83), pregabalin (ROM = 0.59; 95%CI = 0.42–0.84), and valproate (ROM = 0.61; 95%CI = 0.49–0.76) significantly reduced pain intensity. Several other agents also showed significant effects on pain intensity. The network diagram and forest plots are shown in Figure 3. Based on P-scores, the top three interventions were valproate plus magnesium, topiramate, and topiramate plus amitriptyline (Supplementary Table S2). No significant publication bias was detected (Supplementary Figure S10). Pairwise comparisons and network inconsistency tests are detailed in Supplementary Table S6 and Supplementary Figure S11, respectively. The heat map for pain intensity is shown in Supplementary Figure S12.

Valproate plus magnesium and topiramate plus amitriptyline showed the most pronounced effects on pain reduction. These findings support the long-term prophylactic benefits of these interventions and highlight the potential value of combination therapies in migraine management.

### Migraine duration

**FIGURE 4:**
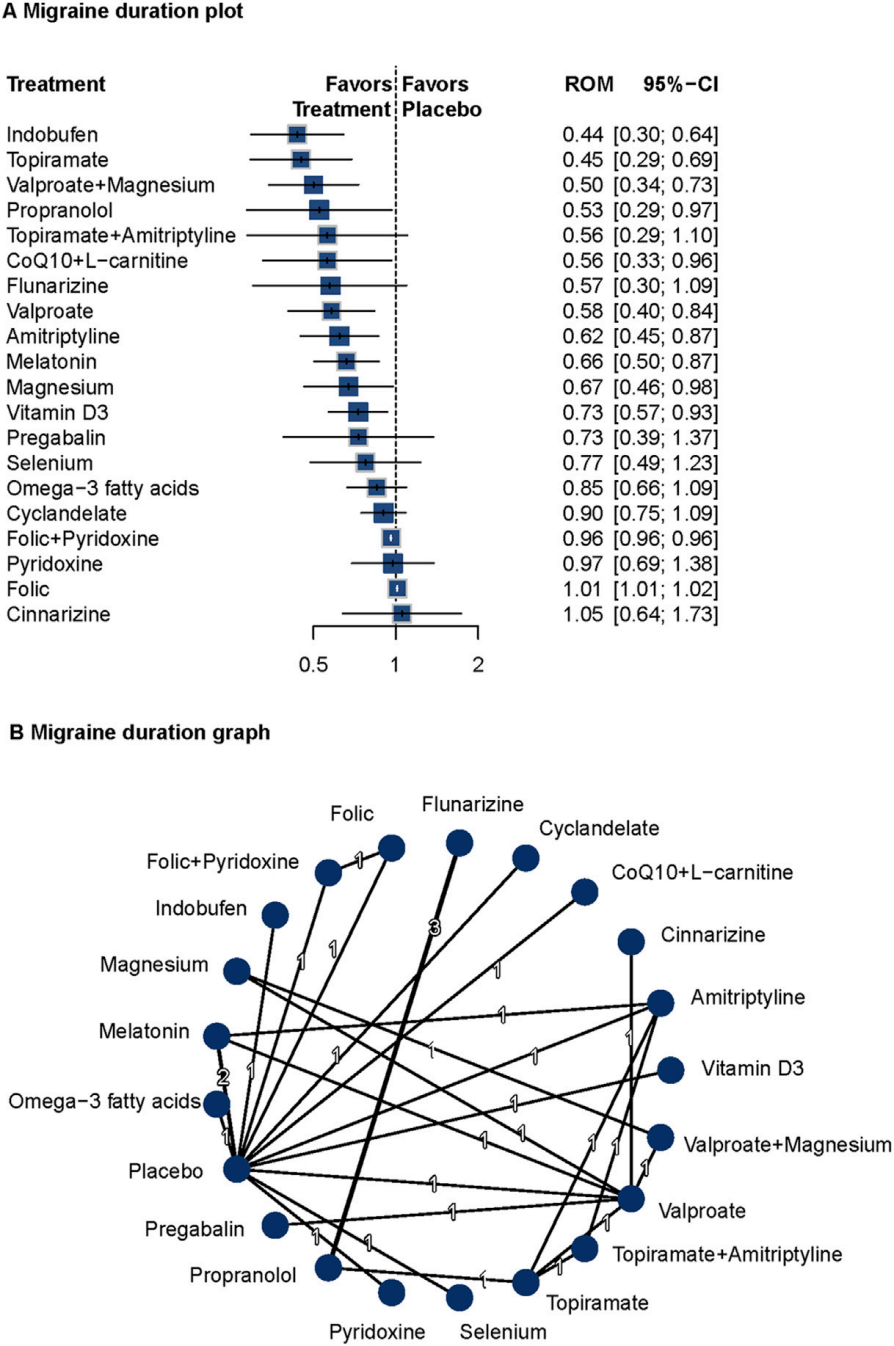
Association of Treatment With Headache Duration. **(A)** Migraine duration plot. **(B)** Migraine duration graph. Forest plot **(A)** and network graph **(B)** displaying the effect of treatments on the duration of migraine attacks. The data includes the ratio of means (RoM) for each treatment compared with placebo, calculated using a fixed-effects model due to heterogeneity (I^2^ = 0%). Significant reductions were observed with indobufen, topiramate, volproate with magnesium, propranolol, CoQ10 with l-carnitine, valproate, amitriptyline, melatonin, magnesium, and vitamin d3. The analysis was based on 18 studies (Carrieri et al., 1988; Gawel et al., 1992; Lucking et al., 1988; Diener et al., 2001a; Diener et al., 2001b; Ashtari et al., 2008; Afshari et al., 2011; Bostani et al., 2013; Sadeghi et al., 2015; Hesami et al., 2018a; Hajihashemi et al., 2019; Ghorbani et al., 2020; Balali et al., 2024; Keskinbora and Aydinli, 2008; Gonçalves et al., 2016; Askari et al., 2017; Ebrahimi-Monfared et al., 2017; Khani et al., 2021) with 1809 patients.

Eighteen studies (n = 1809) reported data on migraine duration (Carrieri et al., 1988; Gawel et al., 1992; Lucking et al., 1988; Diener et al., 2001a; Diener et al., 2001b; Ashtari et al., 2008; Afshari et al., 2011; Bostani et al., 2013; Sadeghi et al., 2015; Hesami et al., 2018a; Hajihashemi et al., 2019; Ghorbani et al., 2020; Balali et al., 2024; Keskinbora and Aydinli, 2008; Gonçalves et al., 2016; Askari et al., 2017; Ebrahimi-Monfared et al., 2017; Khani et al., 2021). A fixed-effects model was used due to low heterogeneity (I^2^ = 0%, Q = 2.55, P = 0.64). Versus placebo, indobufen (ROM = 0.44; 95%CI = 0.30–0.64), topiramate (ROM = 0.45; 95%CI = 0.29–0.69), valproate plus magnesium (ROM = 0.50; 95%CI = 0.34–0.73), propranolol (ROM = 0.53; 95%CI = 0.29–0.97), CoQ10 plus L-carnitine (ROM = 0.56; 95%CI = 0.33–0.96), valproate (ROM = 0.58; 95%CI = 0.40–0.84) were related to decreased migraine duration. Several other agents also showed significant effects. The network diagram and forest plots are shown in Figure 4. P-score rankings are summarized in Supplementary Table S2. No significant publication bias was detected (Supplementary Figure S13). Pairwise comparisons and network inconsistency tests are detailed in Supplementary Table S7 and Supplementary Figure S14, respectively. The heat map for migraine duration is presented in Supplementary Figure S15.

Indobufen, topiramate, and valproate plus magnesium showed significant efficacy in reducing migraine duration. These findings support the long-term prophylactic benefits of these interventions and highlight the potential value of multi-target approaches in migraine prevention.

#### QoL

**FIGURE 5:**
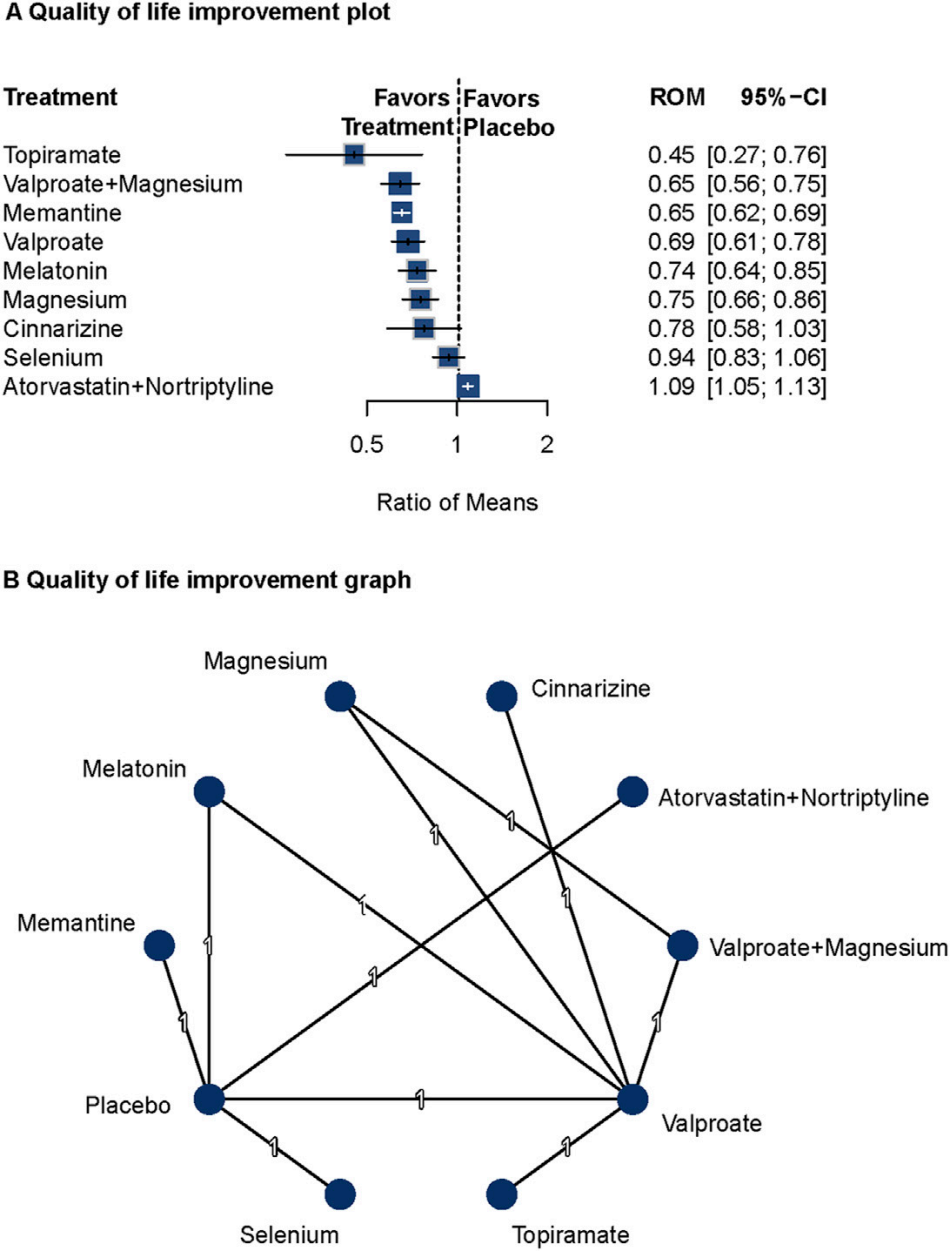
Association of Treatment With Quality of Life. **(A)** Quality of life improvement plot. **(B)** Quality of life improvement graph. Forest plot **(A)** and network graph **(B)** displaying the association of various treatments with quality of life, measured using the Migraine Disability Assessment tool. Topiramate, valproate with magnesium, memantine, valproate, melatonin, and magnesium showed significant improvements in quality of life compared with placebo. The analysis faced limitations due to the small number of studies per treatment.

Seven studies reported data on health-related QoL and disability (Afshari et al., 2011; Bostani et al., 2013; Noruzzadeh et al., 2016; Sherafat et al., 2022; Balali et al., 2024; Ebrahimi-Monfared et al., 2017; Khani et al., 2021). A fixed-effects model was used based on low heterogeneity (Q = 0; I^2^ not reported). Compared with placebo, Topiramate (ROM = 0.45; 95%CI = 0.27–0.76), valproate plus magnesium (ROM = 0.65; 95%CI = 0.56–0.75), memantine (ROM = 0.65; 95%CI = 0.62–0.69), valproate (ROM = 0.69; 95%CI = 0.61–0.78), melatonin (ROM = 0.74; 95%CI = 0.64–0.85), and magnesium (ROM = 0.75; 95%CI = 0.66–0.86) were related to improved QoL. The network diagram and forest plots are shown in Figure 5. No publication bias was observed (Supplementary Figure S16). Pairwise comparisons are detailed in Supplementary Table S8.

Topiramate, valproate plus magnesium, memantine, valproate, melatonin, and magnesium showed significant effects on improving health-related quality of life in patients with migraine. However, due to the limited number of studies, these findings should be interpreted with caution.

#### Safety

Twenty-eight studies (n = 3,090) reported data on adverse events (Carrieri et al., 1988; Steiner et al., 1988; Gawel et al., 1992; Schoenen et al., 1999; Bussone et al., 1999; Diener et al., 2001a; Diener et al., 2001b; Silvestrini et al., 2003; Mei et al., 2004; Sandor et al., 2005; Silberstein et al., 2006; Ashtari et al., 2008; Afshari et al., 2011; Bostani et al., 2013; Sadeghi et al., 2015; Assarzadegan et al., 2016; Noruzzadeh et al., 2016; Hesami et al., 2018a; Shanmugam et al., 2019; Chowdhury et al., 2022; Hedayat et al., 2022; Balali et al., 2024; Mehramiri et al., 2024; Roghani et al., 2024; Diener et al., 1996; Keskinbora and Aydinli, 2008; Luo et al., 2012; Gonçalves et al., 2016). A fixed-effects model was used due to low heterogeneity (I^2^ = 22.8%, Q = 12.96, P = 0.23). Topiramate (RR = 1.43; 95%CI = 1.23–1.65), flunarizine (RR = 1.47; 95%CI = 1.01–2.15), propranolol (RR = 1.53; 95%CI = 1.09–2.13), valproate (RR = 2.09; 95%CI = 1.36–3.22), amitriptyline (RR = 2.12; 95%CI = 1.59–2.83), cinnarizine (RR = 2.27; 95%CI = 1.36–3.81), and nortriptyline (RR = 2.93; 95%CI = 1.39–6.16) were associated with higher rates of adverse events compared with placebo. The forest plots and network diagrams are shown in Supplementary Figure S17, S18. No significant publication bias was detected (Supplementary Figure S19). Pairwise comparisons are detailed in Supplementary Table S9, and network inconsistency tests are shown in Supplementary Figure S20. The heat map for adverse events is presented in Supplementary Figure S21.

Topiramate, valproate, and propranolol were significantly linked to higher rates of adverse events than placebo. These results underscore the need to carefully assess the risk–benefit ratio in migraine prophylaxis.

## Discussion

In this NMA, we evaluated oral medications for migraine prevention. Topiramate, propranolol, and valproate significantly reduced migraine frequency, intensity, and duration, consistent with the 2019 European Headache Federation guidelines, which classify topiramate and propranolol as Level A recommendations and valproate as Level B (Sacco et al., 2019). These agents are also recognized as first-line options in the 2024 International Headache Society Global Practice Recommendations, which synthesize existing evidence on established oral therapies alongside emerging biologics (Puledda et al., 2024a). Despite their efficacy, these medications are associated with higher rates of adverse events, highlighting the need for careful risk–benefit assessment in clinical practice. This study supports the potential role of memantine, melatonin, and vitamin D3 in the prevention of migraine. Although these drugs are not traditionally considered preventive medications, their multi-target mechanisms offer a broader range of therapeutic options for specific patient populations. Specifically, memantine may alleviate migraine attacks by modulating the glutamate signaling pathway, thereby reducing neuronal hyperexcitability (Charles, 2021). Melatonin may indirectly reduce the frequency and pain intensity by regulating circadian rhythms and improving sleep quality (Ong et al., 2018; Nagtegaal et al., 1998). Vitamin D3 may demonstrate significant efficacy in improving migraine by modulating neurotransmitter levels and reducing inflammatory responses (Tseng et al., 2024). However, it is important to note that evidence for these agents is primarily derived from small or single studies, which limits the generalizability of these findings. Therefore, future studies should further explore the mechanisms of action and clinical efficacy of these drugs to verify their long-term effects and safety in migraine prevention.

In addition, this study systematically evaluated the efficacy and safety of various combination therapies. The results showed that flunarizine plus topiramate, valproate plus magnesium, and folic plus pyridoxine were significantly more effective in reducing the frequency, intensity, and duration of migraine attacks compared to monotherapy, and were associated with favorable safety profiles. These combination therapies may generate synergistic effects through distinct mechanisms of action, thus enhancing their preventive efficacy. For example, the combination of valproate and magnesium may enhance the antimigraine effects of valproate by supplementing intraneuronal magnesium ion levels (Khani et al., 2021). Due to the limitations of current network meta-analysis models, the specific contribution of each drug in combination therapies cannot be clearly distinguished. While valproate, topiramate, and amitriptyline are well-established first-line treatments, the added value of adjuncts like magnesium remains uncertain. However, some of these combinations were supported by limited evidence, and larger trials are needed to confirm these findings. Future studies should further investigate the long-term effects and potential side effects of these combination therapy regimens, particularly their efficacy and safety across different patient populations. Investigating the long-term impact of these combination therapies on patients’ QoL, as well as determining the optimal dosage and treatment duration, will be important directions for future research.

In the analysis of the 50% response rate, moderate heterogeneity was observed (I^2^ = 72.7%), and a random-effects model was used to account for between-study variability. Excluding studies with less than 16 weeks of follow-up (Silberstein et al., 2006; Afshari et al., 2011; Bostani et al., 2013; Choudhary et al., 2017) reduced heterogeneity (I^2^ = 50.7%). In contrast, low heterogeneity (I^2^ < 25%) was found for migraine frequency, duration, pain intensity, and quality of life (QoL), supporting the use of a fixed-effect model for these outcomes. The consistency across studies for these endpoints suggests reliable results. The heterogeneity in the 50% response rate may be influenced by follow-up duration; therefore, future studies should standardize follow-up periods to improve comparability. This study found that topiramate, flunarizine, propranolol, valproate, amitriptyline, cinnarizine, and nortriptyline were associated with improved QoL. However, given that the evidence is derived from a limited number of studies, these findings should be interpreted cautiously in clinical practice. Although drugs such as valsartan and a-dihydroergocryptine have shown therapeutic effects, these findings are largely based on individual studies and require further investigation to be confirmed.

34.7% of the participants were from Iran, which may introduce regional bias and limit the generalizability of our findings. Although the included Iranian studies were methodologically sound and met our inclusion criteria, differences in demographics, genetics, or healthcare practices compared to other populations may affect the external validity. Future studies should include more diverse populations to confirm the applicability of these findings across different regions and clinical contexts.

Rimegepant and Atogepant have been approved for migraine prevention (Tepper, , 2020; Deeks, 2022). However, our systematic literature review found no RCTs focusing on these drugs for migraine prevention in patients aged 18–65 years. Some studies have covered a broader age range, such as 18–80 years (Schwedt et al., 2022). This suggests that these drugs may have significant clinical value for a wider patient population. Focusing on patients aged 18–65 years may limit the applicability of our findings. Future research should include a broader age range, such as individuals aged 18 and above, for a more comprehensive perspective. It should also focus on age-stratified studies to better understand the drugs’ therapeutic effects and tolerability.

This study not only validated the efficacy of traditional drugs such as topiramate, valproate, and propranolol, but also focused on uncovering the potential value of non-traditional preventive medications and combination therapies. These agents and combination strategies offer novel approaches to migraine prevention, especially for patients who cannot tolerate conventional therapies. Future studies should further investigate the mechanisms of action and clinical efficacy of these agents to validate their long-term effectiveness and safety in migraine prophylaxis. In addition, given the multi-target nature of these drugs, exploring differences in their efficacy across various patient subgroups will be of considerable importance. QoL measures should be more comprehensively incorporated into future studies to better evaluate the impact of treatment on patients’ daily functioning and overall wellbeing.

## Limitations

Although this study comprehensively evaluated the effects of various oral medications on migraine prevention in adults, it has several limitations. First, variations in the initial measurements of certain endpoints (e.g., frequency, intensity, MIDAS score, and duration) may have affected the reliability of the findings. Second, the validity of the NMA was limited by the inclusion of some medications tested in fewer than 100 patients, which may have introduced small-study bias and influenced the reported results. Third, the evaluation of outcomes such as QoL was based on a restricted number of studies, leading to insufficient statistical power. Fourth, Iran contributed the largest proportion of participants (approximately 34.7% of the total 4,612), which may represent an additional potential source of regional bias. Fifth, this study did not include newer oral preventive agents such as rimegepant and atogepant, which have recently been approved for migraine prevention. This was primarily due to the limited number of RCTs in the predefined age group (18–65 years). These drugs represent emerging treatment options and should be considered in future comparative studies with broader inclusion criteria. In addition, some medications included in this analysis, such as indobufen and cinnarizine, are not approved by the US Food and Drug Administration (FDA), although they have been commonly prescribed in several countries outside the US.

## Conclusion

This network meta-analysis confirms the significant efficacy of traditional agents such as topiramate, valproate, and propranolol in reducing migraine frequency, pain intensity, and duration. However, these medications are associated with a higher incidence of adverse events, highlighting the need for careful risk–benefit assessments. Additionally, the study found that non-traditional medications such as memantine, melatonin, and vitamin D3 may have potential therapeutic effects. Combination therapies, such as flunarizine plus topiramate and valproate plus magnesium, demonstrate superior efficacy and a lower incidence of adverse events compared to monotherapy. When traditional treatments are not tolerated or prove ineffective, combination therapies or non-traditional medications may be considered. For high-risk patients, treatment decisions should prioritize drug safety. Future research should further evaluate the long-term effectiveness and safety of non-traditional preventive medications and combination therapies, particularly in different patient subgroups and in relation to QoL. Overall, this study provides important support for clinical decision-making and highlights potential directions for future research on migraine prevention.

## Data Availability

The original contributions presented in the study are included in the article/Supplementary Material, further inquiries can be directed to the corresponding author.
