# Supplementary material for: Oral preventive medications for migraine in adults aged 18–65: a network meta-analysis
Source: Front Pharmacol. 2025 Sep 4;16:1620887. doi: 10.3389/fphar.2025.1620887 (PMC12443741; doi:10.3389/fphar.2025.1620887)
Supplement: Supplementary file 1 [file Supplementaryfile1.docx]

**Supplemental Online Content**

Supplement to:Preventive Medications in Adult Migraine:A Network Meta-Analysis

Jianping Wu, Jun Wu, Jian Zhang, Bingbing Liu, Xinyuan Song, Yuanjie Wu, Fanian Tian, Yanbing Ding, Ping wang

eAppendix1. PRISMA Checklist

eAppendix2.Search Terms

eFigure 1. PRISMA Flow Diagram Presenting Study Selection Process

eTable 1. Characteristics of Included Studies

eFigure 2. Details of Risk of Bias Assessment for Each Included Study Based on Cochrane Risk of Bias Tool Version 2

eFigure 3. Overall Risk of Bias Percentage for Different Domains Based on Cochrane Risk of Bias Tool Version 2

eTable 2. P-scores of Efficacies of All Study Interventions Included for Each Outcome

eFigure 4. Funnel Plot For Frequency

eTable 3. Network League for Migraine Frequency

eFigure 5. Network Splitting Analysis of Migraine Frequency

eFigure 6. Network Meta-Analysis Heatmap for Migraine Frequency

eTable 4. Covariate Meta-Regression Analysis of Migraine Frequency

eFigure 7. Funnel Plot for 50% Migraine Frequency Reduction Rate

eTable 5. Network League for 50% Migraine Reduction Rate

eFigure 8. Net Splitting Analysis for 50% Migraine Frequency Reduction Rate

eFigure 9. Network Meta-Analysis Heatmap for 50% Migraine Frequency Reduction Rate

eFigure 10. Funnel Plot for Pain Intensity

eFigure 11. Network Splitting Analysis for Pain Intensity

eTable 6. Network League Table for Pain Intensity

eFigure 12. Network Meta-Analysis Heatmap for Pain Intensity

eFigure 13. Funnel Plot for Migraine Duration

eFigure 14. Network Splitting Analysis for Migraine Duration

eTable 7. Network League Table for Migraine Duration

eFigure 15. Network Meta-Analysis Heatmap for Migraine Duration

eFigure 16. Funnel Plot for Quality-of-Life Outcome

eTable 8. Network League Table for Quality of Life

eFigure 17. Forest Plot of Adverse Events of Each Intervention Compared With Placebo

eFigure 18. Network Graph for Adverse Events

eFigure 19. Funnel Plot for Safety

eTable 9. Network League Table for Adverse Events

eFigure 20. Net Splitting Analysis for Adverse Events

eFigure 21. Network Meta-Analysis Heatmap for Adverse Events

eReferences.

This supplemental material has been provided by the authors to give readers additional information about their work.

eAppendix1. PRISMA Checklist

| **Section and Topic** | **Item #** | **Checklist item** | **Location where item is reported** |
| --- | --- | --- | --- |
| **TITLE** | | |  |
| Title | 1 | Identify the report as a systematic review. | Page 1 |
| **ABSTRACT** | | |  |
| Abstract | 2 | See the PRISMA 2020 for Abstracts checklist. | Page 4 |
| **INTRODUCTION** | | |  |
| Rationale | 3 | Describe the rationale for the review in the context of existing knowledge. | Page 6 |
| Objectives | 4 | Provide an explicit statement of the objective(s) or question(s) the review addresses. | Page 6 |
| **METHODS** | | |  |
| Eligibility criteria | 5 | Specify the inclusion and exclusion criteria for the review and how studies were grouped for the syntheses. | Page 7 |
| Information sources | 6 | Specify all databases, registers, websites, organisations, reference lists and other sources searched or consulted to identify studies. Specify the date when each source was last searched or consulted. | Page 7 |
| Search strategy | 7 | Present the full search strategies for all databases, registers and websites, including any filters and limits used. | eAppendix1 |
| Selection process | 8 | Specify the methods used to decide whether a study met the inclusion criteria of the review, including how many reviewers screened each record and each report retrieved, whether they worked independently, and if applicable, details of automation tools used in the process. | Page 7 |
| Data collection process | 9 | Specify the methods used to collect data from reports, including how many reviewers collected data from each report, whether they worked independently, any processes for obtaining or confirming data from study investigators, and if applicable, details of automation tools used in the process. | Page 8 |
| Data items | 10a | List and define all outcomes for which data were sought. Specify whether all results that were compatible with each outcome domain in each study were sought (e.g. for all measures, time points, analyses), and if not, the methods used to decide which results to collect. | Page 8 |
|  | 10b | List and define all other variables for which data were sought (e.g. participant and intervention characteristics, funding sources). Describe any assumptions made about any missing or unclear information. | eTable 1 |
| Study risk of bias assessment | 11 | Specify the methods used to assess risk of bias in the included studies, including details of the tool(s) used, how many reviewers assessed each study and whether they worked independently, and if applicable, details of automation tools used in the process. | Page 8 |
| Effect measures | 12 | Specify for each outcome the effect measure(s) (e.g. risk ratio, mean difference) used in the synthesis or presentation of results. | Page 9 |
| Synthesis methods | 13a | Describe the processes used to decide which studies were eligible for each synthesis (e.g. tabulating the study intervention characteristics and comparing against the planned groups for each synthesis (item #5)). | Page 9 |
|  | 13b | Describe any methods required to prepare the data for presentation or synthesis, such as handling of missing summary statistics, or data conversions. | Page 8-9 |
|  | 13c | Describe any methods used to tabulate or visually display results of individual studies and syntheses. | Page 9 |
|  | 13d | Describe any methods used to synthesize results and provide a rationale for the choice(s). If meta-analysis was performed, describe the model(s), method(s) to identify the presence and extent of statistical heterogeneity, and software package(s) used. | Page 9 |
|  | 13e | Describe any methods used to explore possible causes of heterogeneity among study results (e.g. subgroup analysis, meta-regression). | Page 9 |
|  | 13f | Describe any sensitivity analyses conducted to assess robustness of the synthesized results. | Page 9 |
| Reporting bias assessment | 14 | Describe any methods used to assess risk of bias due to missing results in a synthesis (arising from reporting biases). | Page 8 |
| Certainty assessment | 15 | Describe any methods used to assess certainty (or confidence) in the body of evidence for an outcome. | Page 9 |
| **RESULTS** | | |  |
| Study selection | 16a | Describe the results of the search and selection process, from the number of records identified in the search to the number of studies included in the review, ideally using a flow diagram. | eFigure 1 |
|  | 16b | Cite studies that might appear to meet the inclusion criteria, but which were excluded, and explain why they were excluded. | eFigure 1 |
| Study characteristics | 17 | Cite each included study and present its characteristics. | eTable 1 |
| Risk of bias in studies | 18 | Present assessments of risk of bias for each included study. | eFigure 2-3 |
| Results of individual studies | 19 | For all outcomes, present, for each study: (a) summary statistics for each group (where appropriate) and (b) an effect estimate and its precision (e.g. confidence/credible interval), ideally using structured tables or plots. | Figure1 - 5, eFigure 4 - 21, eTable 2 - 9 |
| Results of syntheses | 20a | For each synthesis, briefly summarise the characteristics and risk of bias among contributing studies. | Page 9-14 |
|  | 20b | Present results of all statistical syntheses conducted. If meta-analysis was done, present for each the summary estimate and its precision (e.g. confidence/credible interval) and measures of statistical heterogeneity. If comparing groups, describe the direction of the effect. | Figure1 - 5, eFigure 4 - 21, eTable 2 - 9 |
|  | 20c | Present results of all investigations of possible causes of heterogeneity among study results. | eFigure4,7,10,13,16,19 |
|  | 20d | Present results of all sensitivity analyses conducted to assess the robustness of the synthesized results. | Page 12 |
| Reporting biases | 21 | Present assessments of risk of bias due to missing results (arising from reporting biases) for each synthesis assessed. | Page 9-14 |
| Certainty of evidence | 22 | Present assessments of certainty (or confidence) in the body of evidence for each outcome assessed. | Not applicable |
| **DISCUSSION** | | |  |
| Discussion | 23a | Provide a general interpretation of the results in the context of other evidence. | Page 14-16 |
|  | 23b | Discuss any limitations of the evidence included in the review. | Page 14-16 |
|  | 23c | Discuss any limitations of the review processes used. | Page 14-16 |
|  | 23d | Discuss implications of the results for practice, policy, and future research. | Page 14-16 |
| **OTHER INFORMATION** | | |  |
| Registration and protocol | 24a | Provide registration information for the review, including register name and registration number, or state that the review was not registered. | Page 7 |
|  | 24b | Indicate where the review protocol can be accessed, or state that a protocol was not prepared. | Page 7 |
|  | 24c | Describe and explain any amendments to information provided at registration or in the protocol. | eAppendix2 |
| Support | 25 | Describe sources of financial or non-financial support for the review, and the role of the funders or sponsors in the review. | Page 18 |
| Competing interests | 26 | Declare any competing interests of review authors. | Page 18 |
| Availability of data, code and other materials | 27 | Report which of the following are publicly available and where they can be found: template data collection forms; data extracted from included studies; data used for all analyses; analytic code; any other materials used in the review. | Page 18 |

**eAppendix. Search Terms**

PubMed (via OVID, Search date: November, 2024)

| 1 | "migraine disORders"[MeSH Terms] | 33560 |
| --- | --- | --- |
| 2 | "Migraine without Aura"[MeSH Terms] | 940 |
| 3 | "Migraine with Aura"[MeSH Terms] | 2312 |
| 4 | "Ophthalmoplegic Migraine"[MeSH Terms] | 68 |
| 5 | "migraine*"[Title/Abstract] | 45688 |
| 6 | "Migraine without Aura"[Title/Abstract] | 2258 |
| 7 | "Migraine with Aura"[Title/Abstract] | 2833 |
| 8 | "Ophthalmoplegic Migraine"[Title/Abstract] | 256 |
| 9 | 1 OR 2 OR 3 OR 4 OR 5 OR 6 OR 7 OR 8 | 50128 |
| 10 | "Amitriptyline"[MeSH Terms] | 6925 |
| 11 | "Amitriptyline"[Title/Abstract] | 7651 |
| 12 | "Piperazines"[Title/Abstract] | 1417 |
| 13 | "Therapeutics"[Title/Abstract] | 126321 |
| 14 | "Therapeutics"[MeSH Terms] | 5429206 |
| 15 | "Flunarizine"[Title/Abstract] | 1786 |
| 16 | "Flunarizine"[MeSH Terms] | 1241 |
| 17 | "frovatriptan"[Title/Abstract] | 201 |
| 18 | "frovatriptan"[Supplementary Concept] | 138 |
| 19 | "metoprolol*"[Title/Abstract] | 8050 |
| 20 | "Metoprolol"[MeSH Terms] | 5803 |
| 21 | "Propanolol"[Title/Abstract] | 600 |
| 22 | "Propranolol"[MeSH Terms] | 33196 |
| 23 | "Valerates"[Title/Abstract] | 12 |
| 24 | "valproic*"[Title/Abstract] | 10709 |
| 25 | "Valerates"[MeSH Terms] | 20608 |
| 26 | "valproate"[Title/Abstract] | 11476 |
| 27 | "Valproic Acid"[MeSH Terms] | 14463 |
| 28 | "Epitomax"[Title/Abstract] | 5 |
| 29 | "Topamax"[Title/Abstract] | 115 |
| 30 | "Topiramate"[Title/Abstract] | 5694 |
| 31 | "Topiramate"[MeSH Terms] | 3161 |
| 32 | "prevention"[Title/Abstract] | 772707 |
| 33 | "control"[Title/Abstract] | 3343270 |
| 34 | "prophylaxis"[Title/Abstract] | 121520 |
| 35 | "preventive therapy"[Title/Abstract] | 3944 |
| 36 | "preventive measures"[Title/Abstract] | 31042 |
| 37 | "prevention and control"[Title/Abstract] | 48100 |
| 38 | "Treatment"[Title/Abstract] | 5679425 |
| 39 | "candesartan"[Title/Abstract] | 3035 |
| 40 | 10 OR 11 OR 12 OR 13 OR 14 OR 15 OR 16 OR 17 OR 18 OR 19 OR 20 OR 21 OR 22 OR 23 OR 24 OR 25 OR 26 OR 27 OR 28 OR 29 OR 30 OR 31 OR 32 OR 33 OR 34 OR 35 OR 36 OR 37 OR 38 OR 39 | 12232529 |
| 41 | "Adult"[MeSH Terms] | 8,272,055 |
| 42 | "Adult"[Title/Abstract] OR "adults"[Title/Abstract] OR "grown ups"[Title/Abstract] OR "grownup"[Title/Abstract] OR "grownups"[Title/Abstract] | 1,654,661 |
| 43 | 41 OR 42 | 9,039,934 |
| 44 | "Middle Aged"[MeSH Terms] | 4,915,200 |
| 45 | "middle age"[Title/Abstract] OR "middle aged"[Title/Abstract] | 68,833 |
| 46 | 44 OR 45 | 4,938,215 |
| 47 | "Young Adult"[MeSH Terms] | 1,088,965 |
| 48 | "adult young"[Title/Abstract] OR "prime adult"[Title/Abstract] OR "prime adults"[Title/Abstract] OR "young adults"[Title/Abstract] OR "young adult"[Title/Abstract] | 128,572 |
| 49 | 47 OR 48 | 1,163,550 |
| 50 | 43 OR 46 OR 49 | 9,053,661 |
| 51 | "randomized controlled trial"[Publication Type] | 629,313 |
| 52 | "controlled clinical trial"[Publication Type] | 720,055 |
| 53 | "Placebos"[MeSH Terms] | 40,101 |
| 54 | "placebo*"[Title/Abstract] | 263,538 |
| 55 | "clinical trials as topic"[MeSH Terms:noexp] | 203,988 |
| 56 | "trial"[Title] | 324,117 |
| 57 | "random"[Title/Abstract] | 396,361 |
| 58 | 51 OR 52 OR 53 OR 54 OR 55 OR 56 OR 57 | 1,483,264 |
| 59 | "animals"[MeSH Terms] | 27695085 |
| 60 | "humans"[MeSH Terms] | 22406843 |
| 61 | 59 NOT 60 | 5,288,242 |
| 62 | 9 AND 40 AND 50 AND 58 AND 61 | 2,853 |

Cochrane Library (Search date: November, 2024)

| #1 | MeSH descriptor: [Migraine Disorders] explode all trees | 3,592 |
| --- | --- | --- |
| #2 | (Migraine Disorders):ti,ab,kw OR (Migraine with Aura):ti,ab,kw OR (Migraine without Aura):ti,ab,kw OR (Ophthalmoplegic Migraine):ti,ab,kw OR (Trigeminal Autonomic Cephalalgias):ti,ab,kw | 5,518 |
| #3 | #1 OR #2 | 5,518 |
| #4 | MeSH descriptor: [Topiramate] explode all trees | 695 |
| #5 | (Topiramate):ti,ab,kw | 1687 |
| #6 | MeSH descriptor: [Valproic Acid] explode all trees | 1163 |
| #7 | (valproic acid):ti,ab,kw OR (Valerates):ti,ab,kw OR (Pentanoic Acids):ti,ab,kw | 2000 |
| #8 | MeSH descriptor: [Propranolol] explode all trees | 3499 |
| #9 | (Propranolol):ti,ab,kw | 5488 |
| #10 | MeSH descriptor: [Metoprolol] explode all trees | 1953 |
| #11 | (Metoprolol):ti,ab,kw | 3352 |
| #12 | (frovatriptan):ti,ab,kw | 101 |
| #13 | MeSH descriptor: [Flunarizine] explode all trees | 179 |
| #14 | (Flunarizine):ti,ab,kw | 463 |
| #15 | MeSH descriptor: [Therapeutics] explode all trees | 438683 |
| #16 | (Therapeutics):ti,ab,kw | 5171 |
| #17 | (Piperazines):ti,ab,kw OR (candesartan):ti,ab,kw OR (Treatment):ti,ab,kw OR (prevention and control):ti,ab,kw OR (preventive measures):ti,ab,kw | 1101201 |
| #18 | (preventive therapy):ti,ab,kw OR (prophylaxis):ti,ab,kw OR (control):ti,ab,kw OR (prevention):ti,ab,kw | 766071 |
| #19 | MeSH descriptor: [Amitriptyline] explode all trees | 1404 |
| #20 | (Amitriptyline):ti,ab,kw | 2804 |
| #21 | #4 OR #5 OR #6 OR #7 OR #8 OR #9 OR #10 OR #11 OR #12 OR #13 OR #14 OR #15 OR #16 OR #17 OR #18 OR #19 OR #20 | 1515998 |
| #22 | #3 AND #21 | 4892 |
| #23 | MeSH descriptor: [Adult] explode all trees | 630611 |
| #24 | (adult):ti,ab,kw OR ("grown-ups"):ti,ab,kw OR (grownup):ti,ab,kw OR (grownups):ti,ab,kw OR (adults):ti,ab,kw | 906481 |
| #25 | MeSH descriptor: [Young Adult] explode all trees | 99997 |
| #26 | (adult, young):ti,ab,kw OR (prime adult):ti,ab,kw OR (prime adults):ti,ab,kw OR (young adults):ti,ab,kw OR (young adult):ti,ab,kw | 136525 |
| #27 | MeSH descriptor: [Middle Aged] explode all trees | 416997 |
| #28 | (middle aged):ti,ab,kw OR (middle age):ti,ab,kw | 466741 |
| #29 | #23 OR #24 OR #24 OR #25 OR #26 OR #27 OR #28 | 1027757 |
| #30 | #22 AND #29 | 3060 |

Embase (Search date: November, 2024)

| (('migraine'/exp OR 'migraine') AND [embase]/lim OR (('familial migraine':ti,ab,kw OR 'headache, migrainous':ti,ab,kw OR 'hemicrania':ti,ab,kw OR 'migraine disorders':ti,ab,kw OR 'migrainous headache':ti,ab,kw OR 'status hemicranicus':ti,ab,kw OR 'migraine':ti,ab,kw) AND [embase]/lim)) AND (('prevention and control'/exp OR 'prevention and control') AND [embase]/lim OR (prevention:ti,ab,kw AND control:ti,ab,kw AND [embase]/lim) OR (('prophylaxis'/exp OR 'prophylaxis') AND [embase]/lim) OR 'disease prevention':ti,ab,kw OR 'disease prophylaxis':ti,ab,kw OR 'health protection':ti,ab,kw OR 'prevention, disease':ti,ab,kw OR 'preventive medication':ti,ab,kw OR 'preventive therapy':ti,ab,kw OR 'preventive treatment':ti,ab,kw OR 'prophylactic institution':ti,ab,kw OR 'prophylactic management':ti,ab,kw OR 'prophylactic medication':ti,ab,kw OR 'prophylactic therapy':ti,ab,kw OR 'prophylactic treatment':ti,ab,kw OR 'prophylaxis':ti,ab,kw OR (('prevention'/exp OR 'prevention') AND [embase]/lim) OR (('precautionary action':ti,ab,kw OR 'preventive action':ti,ab,kw OR 'preventive measure':ti,ab,kw OR 'prevention':ti,ab,kw) AND [embase]/lim) OR (('therapy'/exp OR 'therapy') AND [embase]/lim) OR (('combination therapy':ti,ab,kw OR 'disease therapy':ti,ab,kw OR 'disease treatment':ti,ab,kw OR 'diseases treatment':ti,ab,kw OR 'disorder treatment':ti,ab,kw OR 'disorders treatment':ti,ab,kw OR 'efficacy, therapeutic':ti,ab,kw OR 'illness treatment':ti,ab,kw OR 'medical therapy':ti,ab,kw OR 'medical treatment':ti,ab,kw OR 'multiple therapy':ti,ab,kw OR 'polytherapy':ti,ab,kw OR 'somatotherapy':ti,ab,kw OR 'therapeutic action':ti,ab,kw OR 'therapeutic efficacy':ti,ab,kw OR 'therapeutic trial':ti,ab,kw OR 'therapeutic trials':ti,ab,kw OR 'therapeutics':ti,ab,kw OR 'therapy, medical':ti,ab,kw OR 'treatment effectiveness':ti,ab,kw OR 'treatment efficacy':ti,ab,kw OR 'treatment, medical':ti,ab,kw OR 'therapy':ti,ab,kw) AND [embase]/lim) OR (('drug'/exp OR 'drug') AND [embase]/lim) OR (('acid drug':ti,ab,kw OR 'basic drug':ti,ab,kw OR 'biopharmaceutic agent':ti,ab,kw OR 'drugs':ti,ab,kw OR 'medicament':ti,ab,kw OR 'pharmaceutical preparations':ti,ab,kw OR 'pharmaceutical substance':ti,ab,kw OR 'pharmacochemic':ti,ab,kw OR 'pharmacochemical agent':ti,ab,kw OR 'pharmacon':ti,ab,kw OR 'synthetic drug':ti,ab,kw OR 'synthetic drugs':ti,ab,kw OR 'drug':ti,ab,kw) AND [embase]/lim) OR (('topiramate'/exp OR 'topiramate' OR 'valproic acid'/exp OR 'valproic acid' OR 'propranolol'/exp OR 'propranolol' OR 'metoprolol'/exp OR 'metoprolol' OR 'frovatriptan'/exp OR 'frovatriptan' OR 'flunarizine'/exp OR 'flunarizine' OR 'amitriptyline'/exp OR 'amitriptyline') AND [embase]/lim)) AND ('clinical trial':ti,ab,kw OR 'randomized controlled trial':ti,ab,kw OR 'controlled clinical trial':ti,ab,kw OR placebo:ti,ab,kw) AND [embase]/lim AND [humans]/lim AND (('adult'/exp OR 'adult') AND [embase]/lim OR (('adults':ti,ab,kw OR 'grown-ups':ti,ab,kw OR 'grownup':ti,ab,kw OR 'grownups':ti,ab,kw OR 'adult':ti,ab,kw) AND [embase]/lim) OR (('middle aged'/exp OR 'middle aged') AND [embase]/lim) OR (('middle age':ti,ab,kw OR 'middle aged':ti,ab,kw) AND [embase]/lim) OR (('young adult'/exp OR 'young adult') AND [embase]/lim) OR (('adult, young':ti,ab,kw OR 'prime adult':ti,ab,kw OR 'prime adults':ti,ab,kw OR 'young adults':ti,ab,kw OR 'young adult':ti,ab,kw) AND [embase]/lim)) |
| --- |
|  |
|  |
|  |
|  |
|  |
|  |
|  |
|  |
|  |
|  |
|  |
|  |
|  |
|  |

Scopus (Search date: November, 2024)

| ( TITLE-ABS-KEY ( "Migraine" ) ) AND ( ( TITLE-ABS-KEY ( topiramate ) OR TITLE-ABS-KEY ( "valproic acid" ) OR TITLE-ABS-KEY ( valerates ) OR TITLE-ABS-KEY ( propranolol ) OR TITLE-ABS-KEY ( metoprolol ) OR TITLE-ABS-KEY ( frovatriptan ) OR TITLE-ABS-KEY ( flunarizine ) OR TITLE-ABS-KEY ( amitriptyline ) OR TITLE-ABS-KEY ( placebos ) OR TITLE-ABS-KEY ( therapeutics ) ) ) AND ( ( TITLE-ABS-KEY ( "randomized controlled trial" ) OR TITLE-ABS-KEY ( "controlled clinical trial" ) OR TITLE-ABS-KEY ( "random" ) OR TITLE-ABS-KEY ( "clinical trials as topic" ) OR TITLE-ABS-KEY ( trial ) ) ) AND NOT ( ( TITLE-ABS-KEY ( animals ) AND NOT TITLE-ABS-KEY ( humans ) ) ) |
| --- |
|  |
|  |
|  |
|  |
|  |

**eFigure 1. PRISMA Flow Diagram Presenting Study Selection Process**

Identification of studies via other methods

Identification of studies via databases

Studies included inreview(n=44)

Reports excluded:

Acute treatment(n=22)

Irrelevant intervention(n=63)

Design(n=89)

Does study(n=9)

Sample size(n=29)

Age(n=71)

Outcomes(n=9)

Data(n=13)

Reports assessed for eligibility

(n=349)

Reports not retrieved(n=27)

Reports sought for retrieval (n=376)

Records excluded(n=10974)

Records screened (n=11350)

Screening

ldentification

All reports were previously found

in our search

Reports assessed for eligibility

(n =5)

Reports sought for retrieval (n =5)

Records identified

from:Citation

searching(n=5)

Records removed before screening: Duplicate records removed(n=6093)

Records identified from Databases(n=17443)

lncluded

| **eTable 1. Characteristics of Included Studies** This table outlines the key characteristics of the included studies on migraine interventions, including the study author and year, country of origin, total number of participants, migraine characteristics, diagnostic criteria used, intervention details, measured outcomes, overall follow-up duration, and the assessed risk of bias. Outcomes are categorized as follows: Frequency - the number of migraine attacks per month after treatment; 50% responder rate - defined as the number of patients with at least a 50% reduction in migraine frequency after treatment compared to baseline; Intensity- self-reported migraine intensity, a numeric assessment of pain intensity from 0 to 10; Quality of Life/Disability - disability produced by migraine, measured by the MIDAS tool; Duration- the length of each individual migraine attack, measured in minutes or hours. For studies with multiple endpoints, the last follow-up time was selected for analysis. Additionally, the overall number of adverse events was monitored to assess safety. Abbreviations: ICHD - International Classification of Headache Disorders; NR - Not Reported; IU - International Units; MIDAS - Migraine Disability Assessment Questionnaire. The caliber of the filtered studies was evaluated using the Cochrane Risk of Bias Tool, version 2. | | | | | | | | | | | | | | | |
| --- | --- | --- | --- | --- | --- | --- | --- | --- | --- | --- | --- | --- | --- | --- | --- |
|  |  |  |  |  |  |  |  |  |  |  |  |  |  |  |  |
| Study | Year | Country | Total participants | Migraine characteristics | Diagnostic criteria | Intervertions | | | Outcomes | | | | | Overall follow-up | Risk of bias |
|  |  |  |  |  |  | 1 | 2 | 3 | Frequency | 50%Responder rate | Intensity | QoL/Disability | Duration |  |  |
| Carrieri (1) | 1988 | Italy | 35 | NR | Ad Hoc Committee | Indobufen 200mg,bid | Placebo | _ | √ |  |  |  | √ | 3 months | Low |
| Steiner (2) | 1988 | USA | 59 | NR | NR | Metoprolol 50mg or 100mg,bid | Placebo | _ | √ |  | √ |  |  | 12 weeks | Low |
| Stewart (3） | 1988 | Canada | 26 | W & W/O Aura | Neurologist | Nimodipine 40mg,tid | Placebo | _ | √ |  |  |  |  | 4 months | Low |
| Tuca (4) | 1989 | Spain | 30 | NR | NR | Nicardipine 20mg,bid | Placebo | _ |  | √ |  |  |  | 4 months | Mediu m |
| Gawel (5) | 1992 | Canada | 94 | W & W/O Aura | the World Federation of Neurology Research Group in Migraine | Propranolol (Titrated 80mg bid) | Flunarizine (Titrated 10mg,qn) | _ |  |  | √ |  | √ | 5 months | Low |
| Peikert (6) | 1996 | Germany | 81 | W & W/O Aura | IHS | Magnesium | Placebo | _ |  | √ |  |  |  | 16 weeks | Low |
| Schoenen (7) | 1998 | Luxemburg | 54 | NR | IHS | Riboflavin 400 mg,qd | Placebo | _ |  | √ |  |  |  | 4 months | Low |
| Bussone (8) | 1999 | Italy | 135 | without aura | IHS | A-dihydroergocryptine 10mg,bid | Flunarizine 5mg,qd | _ | √ | √ |  |  |  | 7 months | Low |
| Diener (9) | 2001 | Austria,Belgium,Germany and Switzerland | 270 | W & W/O Aura | IHS | Acetylsalicylic acid 100mg,qd | Metoprolol (Titrated 200mg,qd) | _ | √ |  |  |  |  | 20 weeks | Mediu m |
| Diener (10) | 2001 | Germany | 249 | W & W/O Aura | IHS | Cyclandelate 1600mg,qd | Placebo | _ | √ |  |  |  | √ | 24 weeks | Mediu m |
| Pradalier (11) | 2001 | France | 196 | W & W/O Aura | IHS | Omega-3 polyunsaturated fatty acids 3g,bid | Placebo | _ | √ | √ | √ |  | √ | 20 weeks | Mediu m |
| Edwards (12) | 2003 | England | 70 | W & W/O Aura | IHS | Topiramate  (Titration 100mg bid) | Placebo | _ |  | √ |  |  |  | 16 weeks | Mediu m |
| Silvestrini (13) | 2003 | Italy | 28 | NR | ICHD | Topiramate  (Titration 50mg qd) | Placebo | _ | √ | √ |  |  |  | 16 weeks | Mediu m |
| Mei (14) | 2004 | Italy | 72 | W & W/O Aura | IHS | Topiramate 100mg qd | Placebo | _ |  | √ |  |  |  | 20 weeks | Mediu m |
| Sándor (15) | 2005 | Europe | 42 | W & W/O Aura | IHS | CoQ10 | Placebo | _ |  | √ |  |  |  | 4 months | Mediu m |
| Silberstein (16) | 2006 | USA | 211 | W & W/O Aura | IHS | Topiramate  (Titration 100mg bid) | Placebo | _ |  | √ |  |  |  | 12 weeks | Mediu m |
| Ashtari (17) | 2008 | Iran | 62 | W & W/O Aura | IHS | Topiramate  (Titration 50mg qd) | Propranolol (Titrated 80mg bid) | _ | √ |  | √ |  | √ | 8 weeks | Mediu m |
| Keskinbora (18) | 2008 | Turkey | 63 | W & W/O Aura | IHS | Topiramate | Amitriptyline | Topiramate + Amitriptyline | √ |  | √ |  | √ | 12 weeks | Mediu m |
| Afshari (19) | 2012 | Iran | 56 | W & W/O Aura | IHS | Topiramate 50mg/d | Valproate 40mg/d | _ | √ | √ | √ | √ | √ | 12 weeks | Low |
| Bostani (20) | 2013 | Iran | 104 | W & W/O Aura | IHS | Cinnarizine | Sodium valproate | _ | √ | √ | √ | √ | √ | 12 weeks | Low |
| Sadeghi (21) | 2015 | Iran | 54 | With Aura | IHS | Pyridoxine 40mg,bid | Placebo | _ | √ |  | √ |  | √ | 12 weeks | Mediu m |
| Assarzadegan (22) | 2016 | Iran | 89 | W & W/O Aura | IHS | Zonisamide | Placebo | _ | √ |  | √ |  |  | 3 months | Mediu m |
| Goncalves (23) | 2016 | Brazil | 178 | W & W/O Aura | IHS | Amitriptyline | Melatonin | Placebo |  | √ | √ |  | √ | 16 weeks | Low |
| Noruzzadeh (24) | 2016 | Iran | 60 | Without Aura | ICHD-2 | Memantine hydrochloride (Titration 10,mg/d) | Placebo | _ | √ |  | √ | √ |  | 12 weeks | Low |
| Askari (25) | 2017 | Iran | 95 | W & W/O Aura | IHS | Folic acid + Pyridoxine | Folic acid | Placebo | √ |  | √ |  | √ | 3 months | Low |
| Choudhary (26) | 2017 | Pakistan | 90 | NR | NR | Topiramate | Sodium valproate | _ | √ | √ |  |  |  | 6 weeks | Mediu m |
| Ebrahimi-Monfared (27) | 2017 | Iran | 105 | W & W/O Aura | IHS | Sodium Valproate 200mg/d | Melatonin 3,mg | Placebo | √ |  | √ | √ | √ | 2 months | Mediu m |
| Hesami (28) | 2018 | Iran | 88 | W & W/O Aura | ICHD-3 | Valproate 400mg/d | Pregabalin 100mg/d | _ | √ | √ | √ |  | √ | 3 months | Low |
| Gazerani (29) | 2019 | Denmark | 48 | NR | ICHD-2 | Vitamin D3 100 μg/day | Placebo | _ | √ |  | √ |  |  | 28 weeks | Low |
| Hajihashemi (30) | 2019 | Iran | 56 | W & W/O Aura | IHS | CoQ10 + L-carnitine | Placebo | _ | √ |  | √ |  | √ | 8 weeks | Low |
| Shanmugam (31) | 2019 | India | 57 | NR | IHS | Memantine | Placebo | _ | √ | √ |  |  |  | 24 weeks | Mediu m |
| Ghorbani (32) | 2020 | Iran | 74 | NR | ICHD-3 | Vitamin D3 2000 IU/d | Placebo | _ | √ |  | √ |  | √ | 16 weeks | Low |
| Khani (33） | 2021 | Iran | 222 | W & W/O Aura | NR | Magnesiu + Sodium valproate | Sodium valproate | Magnesium | √ |  | √ | √ | √ | 4 months | Mediu m |
| Chowdhury (34) | 2022 | India | 95 | W & W/O Aura | ICHD-3 | Topiramate 100mg/d | Propranolol 160mg/d | _ |  | √ |  |  |  | 28 weeks | Mediu m |
| Sherafat (35) | 2022 | Iran | 68 | NR | IHS | Atorvastati + Nortriptyline | Placebo | _ |  |  | √ | √ |  | 24 weeks | Low |
| Hedayat (36) | 2022 | Iran | 80 | W & W/O Aura | ICHD-3 | Amitriptyline 25mg/d | Venlafaxine 37.5mg/d | _ | √ |  |  |  |  | 8 weeks | Low |
| Balali (37) | 2024 | Iran | 72 | W & W/O Aura | ICHD-3 | Selenomethionine 200mg/d | Placebo | _ | √ |  | √ | √ | √ | 12 weeks | Mediu m |
| Mehramiri (38) | 2024 | Iran | 60 | NR | ICHD-3 | Melatonin + Propranolol | Propranolol | _ |  |  |  |  |  | 2 months | Low |
| Mosarrezaii (39) | 2024 | Iran | 56 | NR | neurologist | Propranolol 20mg,bid | Valsartan 40mg,qd | _ | √ |  |  |  |  | 12 weeks | Low |
| Roghani (40) | 2024 | Iran | 201 | NR | IHS | Venlafaxine 37.5mg,bid | Nortriptyline 25mg,qn | _ | √ |  |  |  |  | 3 months | Low |
| Hesami (41) | 2018 | China | 82 | NR | ICHD-3 | Atorvastatin 40mg/d | Sodium valproate 500mg/d | _ |  | √ |  |  |  | 3 months | Mediu m |
| Diener (42) | 1996 | Europe | 214 | W & W/O Aura | IHS | Cyclandelate | Propranolol | Placebo |  | √ |  |  |  | 16 weeks | Low |
| Lucking (43) | 1988 | Germany | 405 | NR | NR | Flunarizine | Propranolol | _ | √ |  |  |  | √ | 4 months | Mediu m |
| Ning Luo (44) | 2012 | China | 126 | W & W/O Aura | IHS | Flunarizine | Topiramate | Flunarizine + Topiramate | √ | √ |  |  |  | 12 months | Mediu m |

**eFigure 2. Details of Risk of Bias Assessment for Each Included Study Based on Cochrane Risk of Bias Tool Version 2**

**
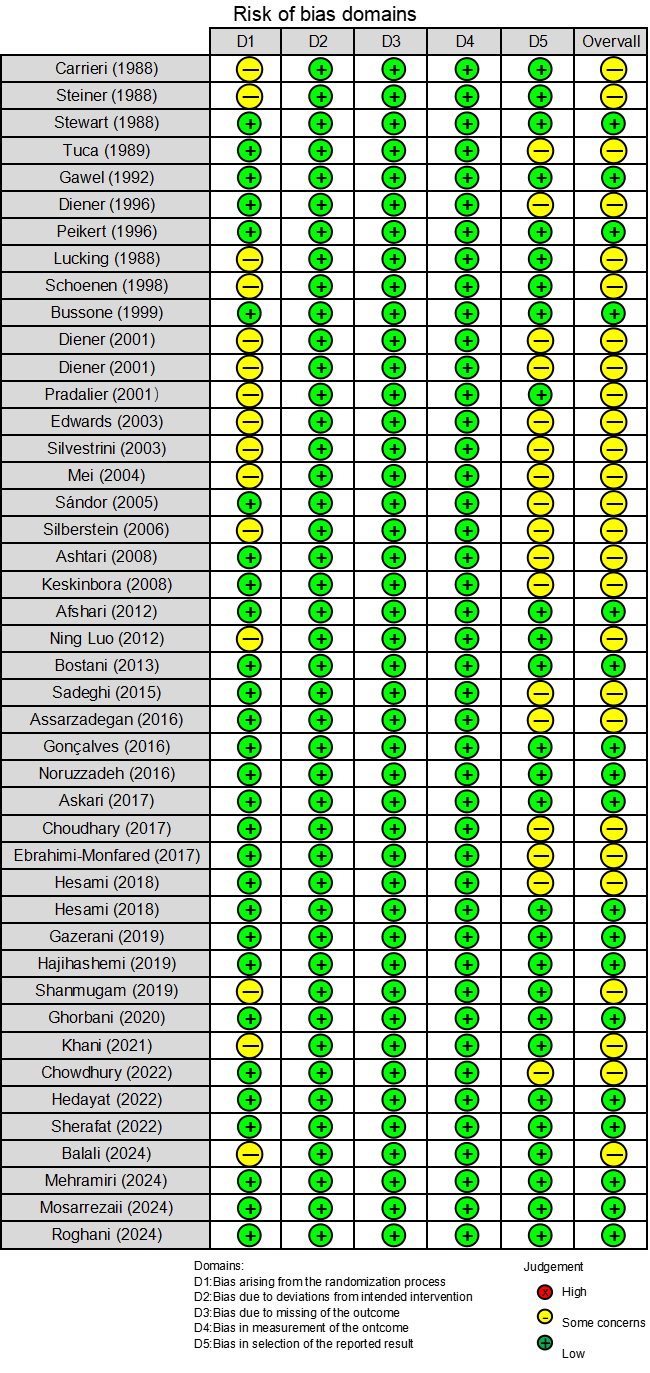
**

**eFigure 3. Overall Risk of Bias Percentage for Different Domains Based on Cochrane Risk of Bias Tool Version 2**

**
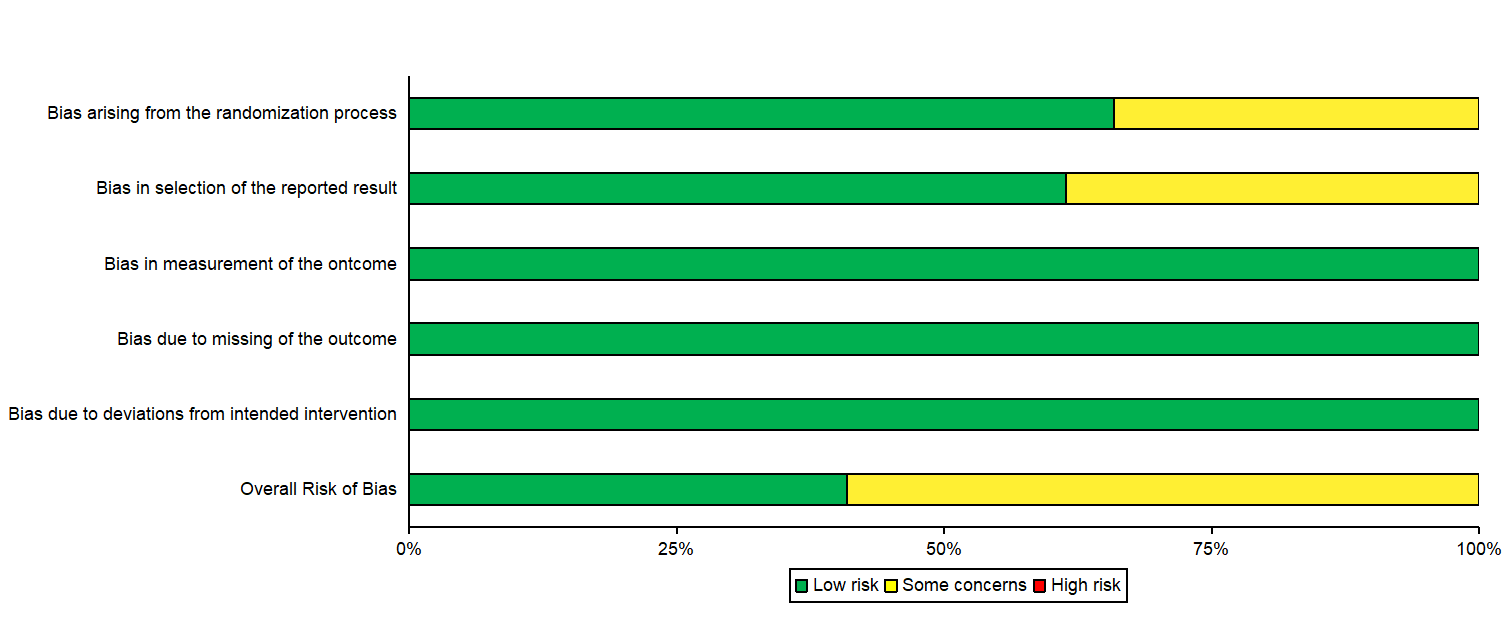
**

**
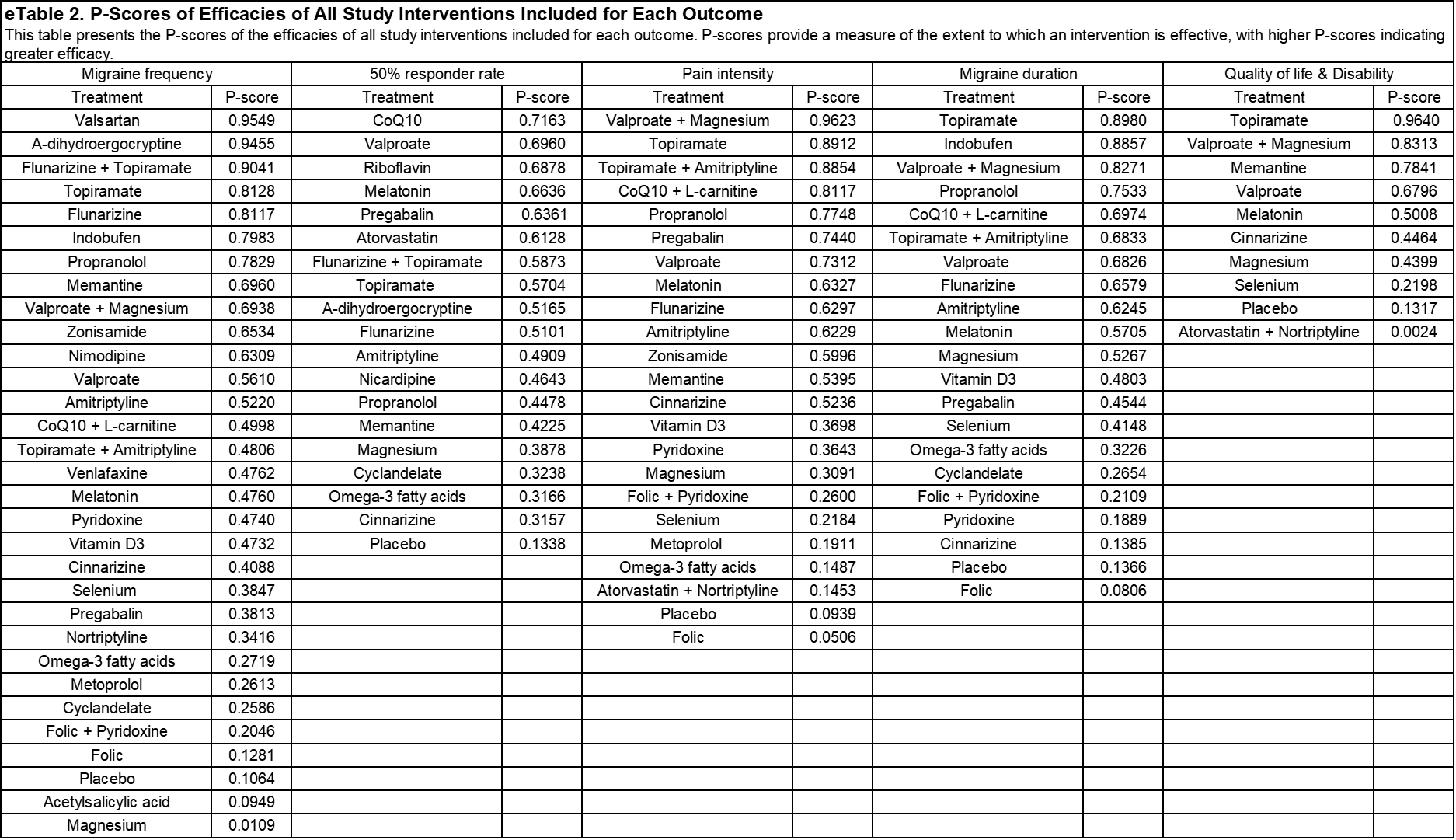
**

eFigure 4. Funnel Plot For Frequency

**

**

**
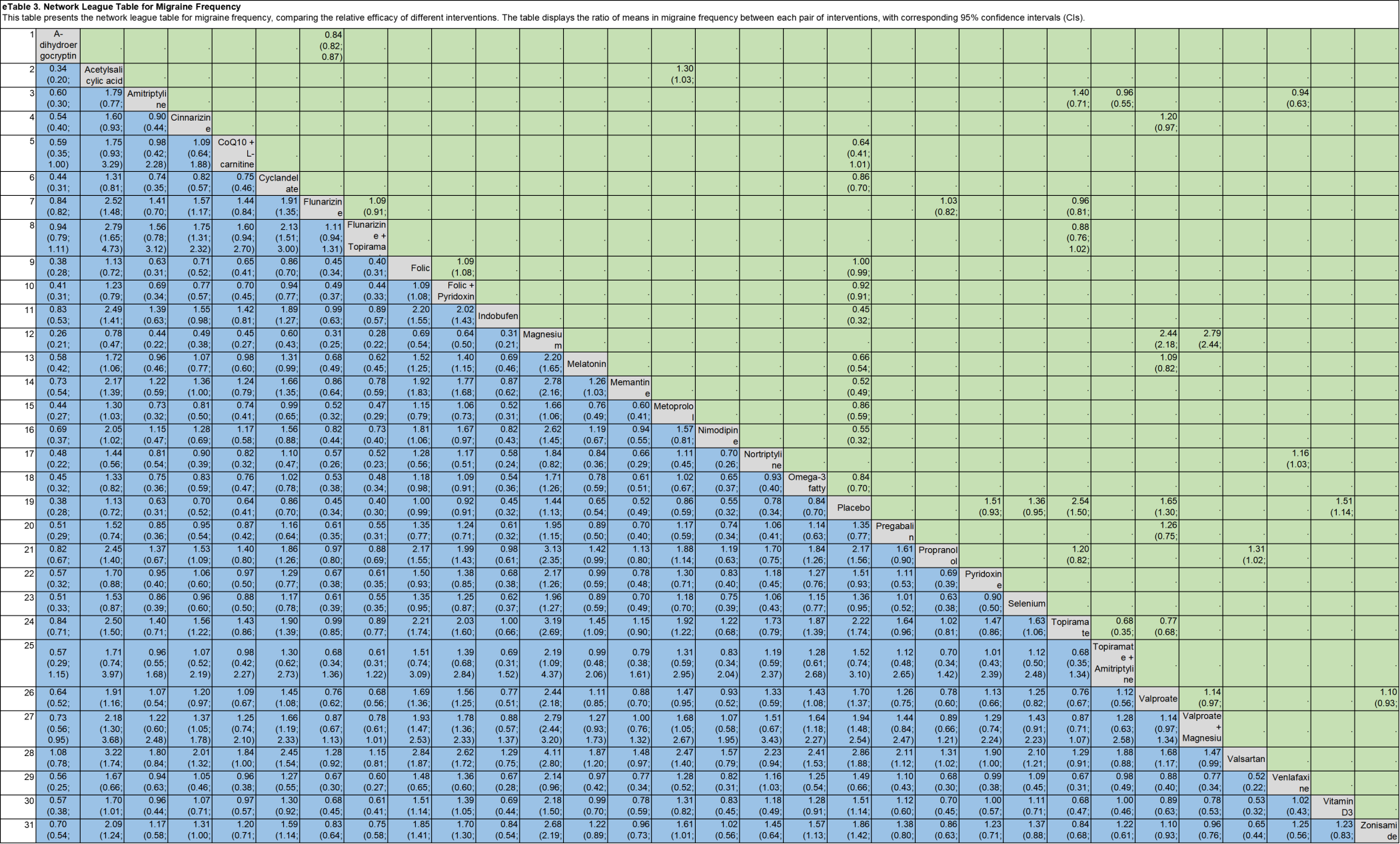
**

**eFigure 5. Network Splitting Analysis of Migraine Frequency**


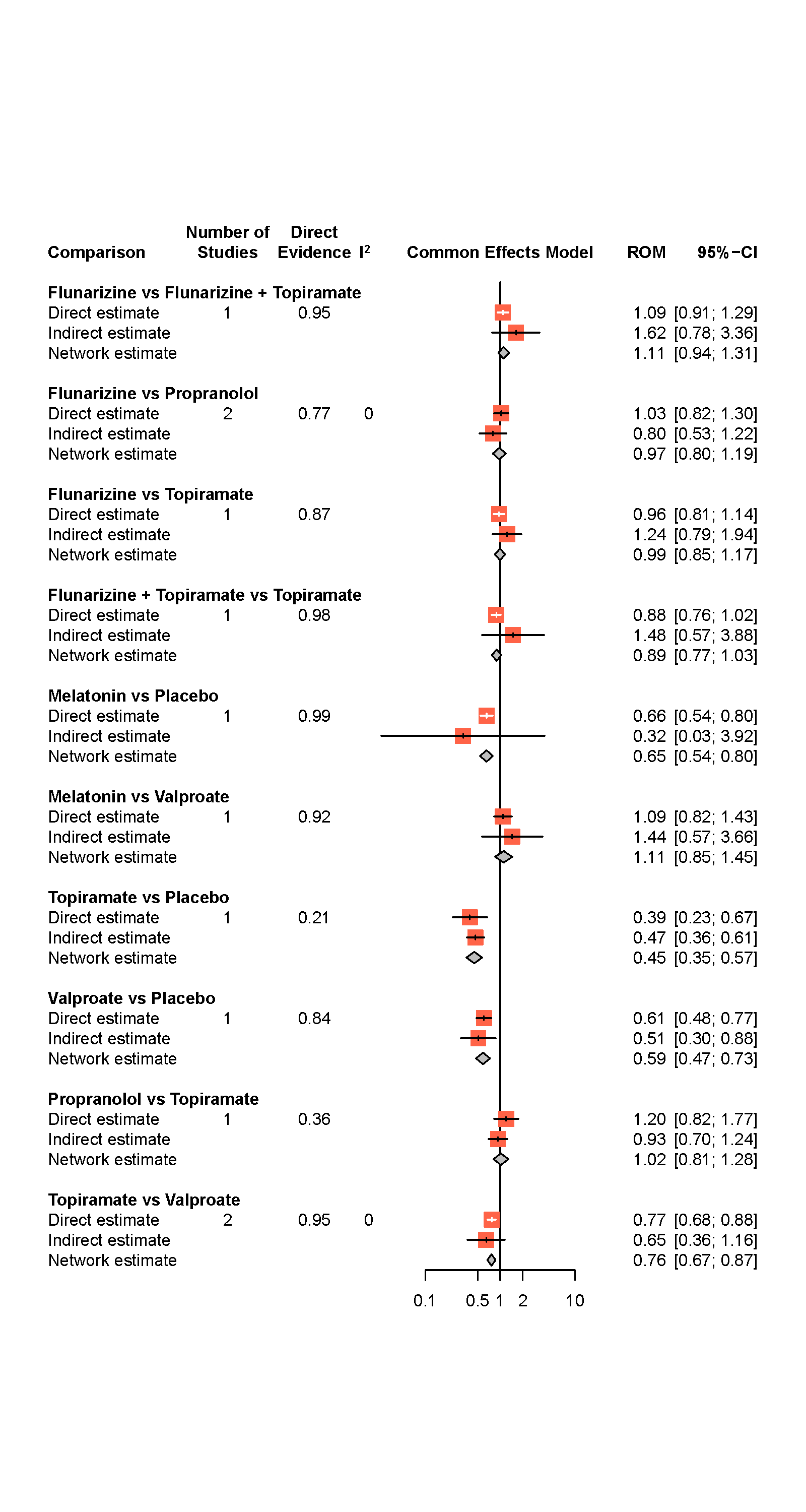


This figure presents the network splitting analysis of migraine frequency, which examines the consistency between direct and indirect evidence within the network meta-analysis. By splitting the network into these two types of evidence, the analysis evaluates whether the conclusions drawn from the meta analysis are consistent regardless of the source of evidence, ensuring the robustness and reliability of the overall results.

**eFigure 6. Network Meta-Analysis Heatmap for Migraine Frequency**


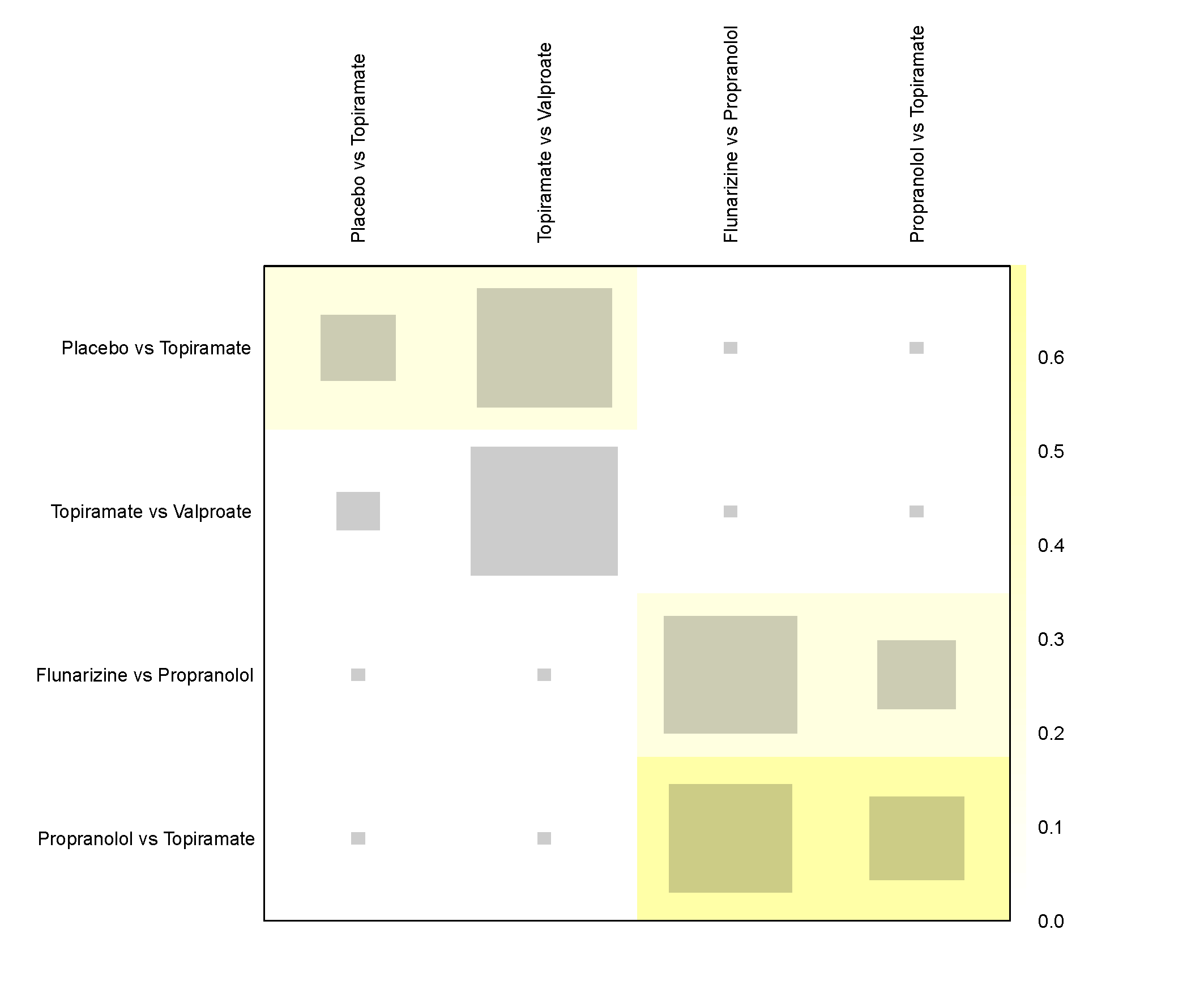


The net heat plot is a matrix visualization that highlights areas of inconsistency within the network - meta analysis. Each gray square's area represents the contribution of the direct estimate from the design in the column to the network estimate in the row. The colors indicate the change in inconsistency when relaxing the consistency assumption for single designs. Diagonal colors show the inconsistency contribution of the corresponding design, whereas off-diagonal colors reflect the change in inconsistency between direct and indirect evidence. Clustering identifies hot spots of inconsistency, helping to locate potential sources for further investigation.

**eTable 4.Covariate Meta-Regression Analysis of Migraine Frequency**

| Covariate | ROM | 95%(CI) | P-value |
| --- | --- | --- | --- |
| Follow-up | 0.984 | (0.92; 1.05) | 0.651 |
| Age | 1.019 | (0.84; 1.24) | 0.850 |
| Migraine with Aura | 1.403 | (0.33; 5.88) | 0.644 |
| Non-ICHD Diagnostic | 0.351 | (0.07; 1.68) | 0.189 |
| proportion of males | 0.003 | (1.59e-06; 4.77) | 0.121 |

**eFigure 7. Funnel Plot for 50% Migraine Frequency Reduction Rate**

**

**

**
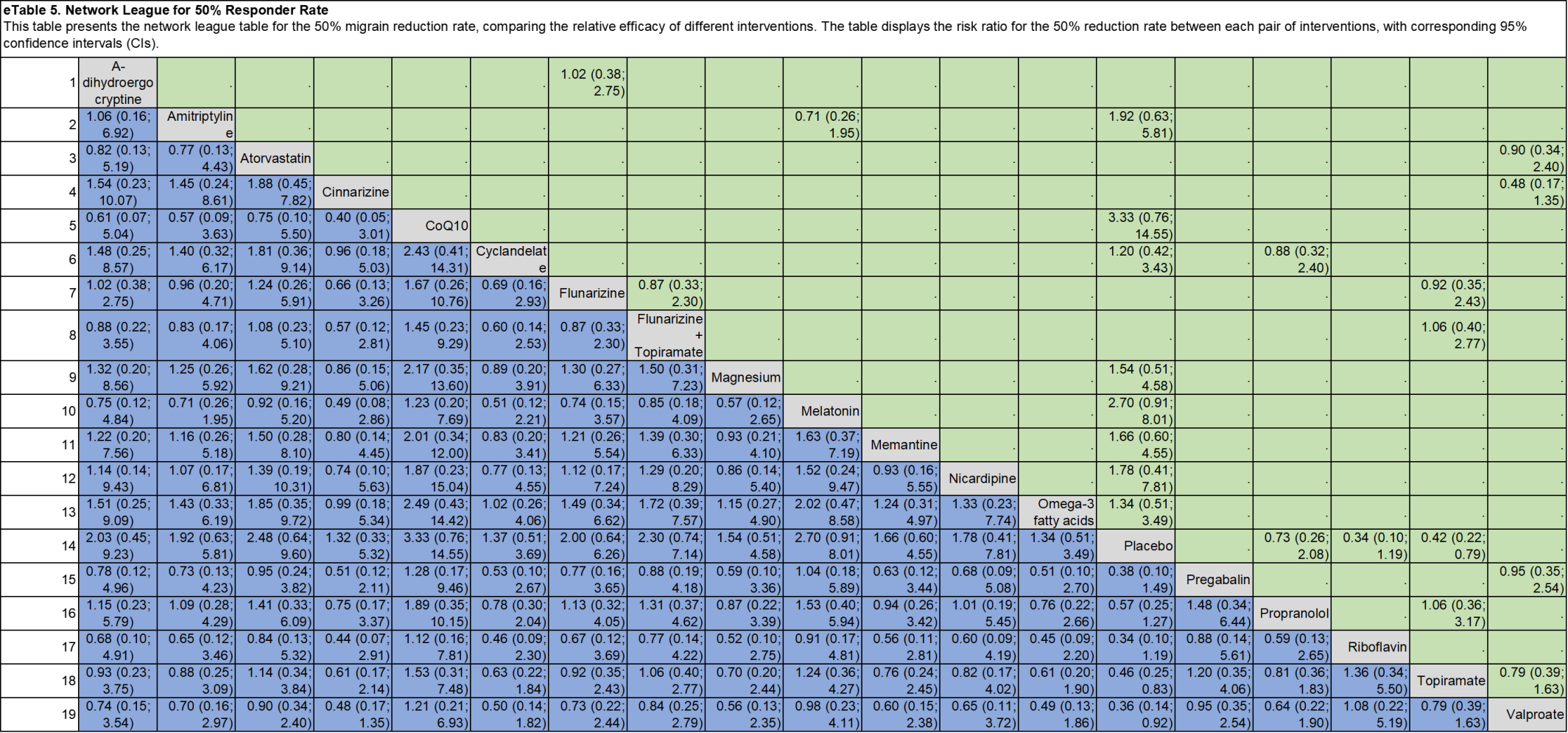
**

**eFigure 8. Net Splitting Analysis for 50% Migraine Frequency Reduction Rate**

**
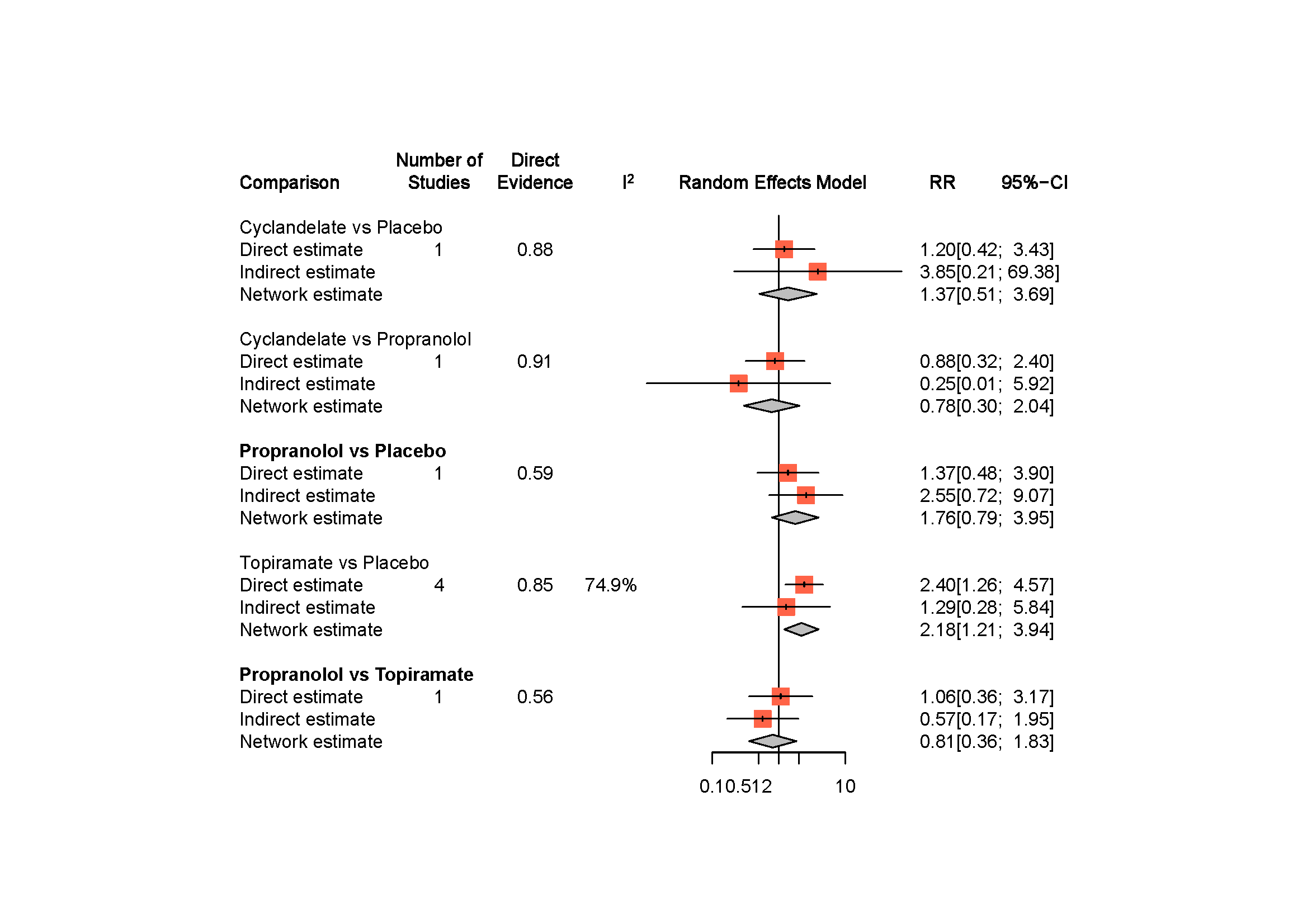
**

This figure presents the network splitting analysis for the 50% migraine frequency reduction rate, which examines the consistency between direct and indirect evidence within the network meta-analysis. By splitting the network into these two types of evidence, the analysis evaluates whether the conclusions drawn from the meta-analysis are consistent regardless of the source of evidence, ensuring the robustness and reliability of the overall results.

**eFigure 9. Network Meta-Analysis Heatmap for 50% Migraine Frequency Reduction Rate**

**
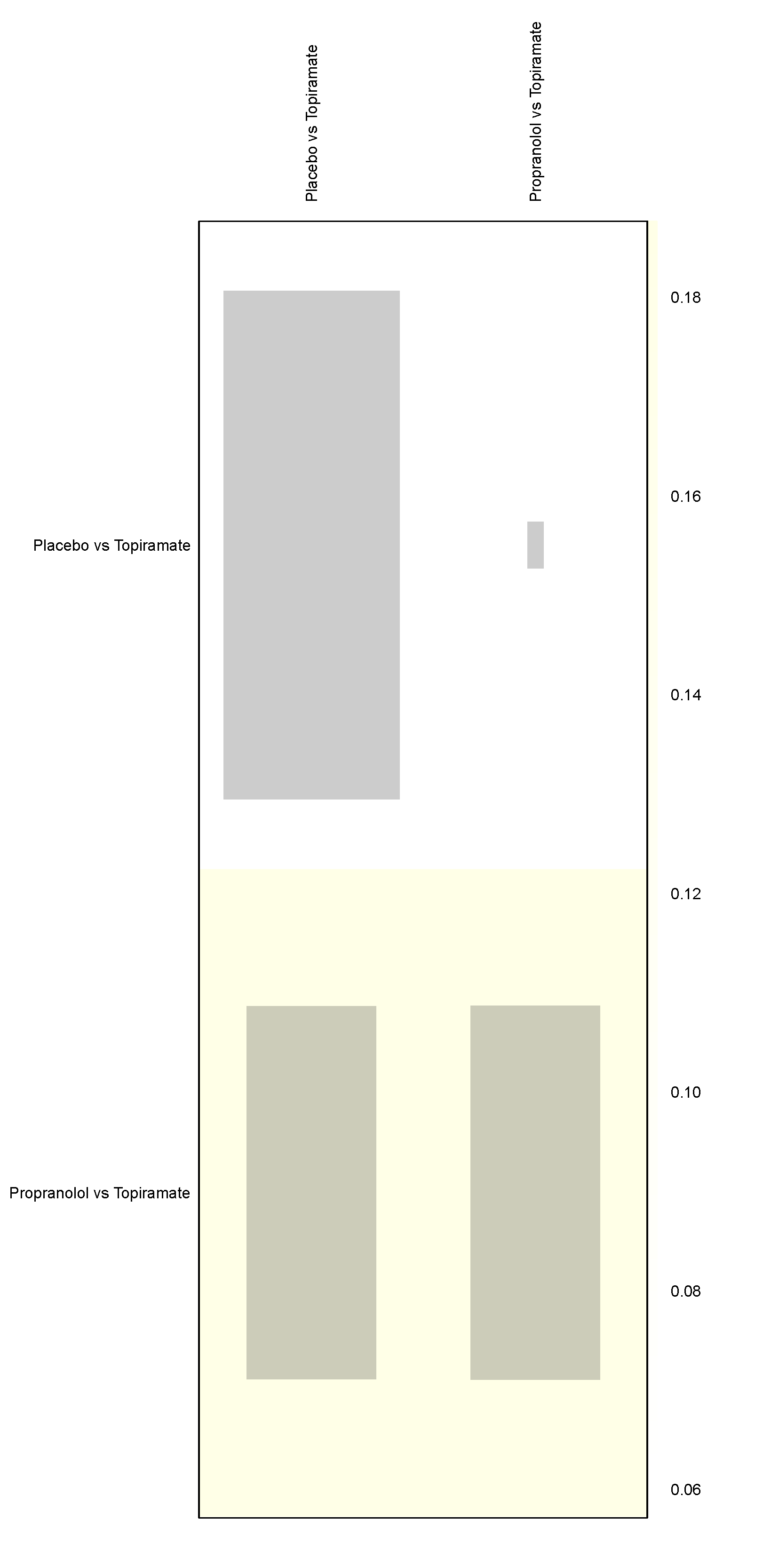
**

The net heat plot is a matrix visualization that highlights areas of inconsistency within the network - meta analysis. Each gray square's area represents the contribution of the direct estimate from the design in the column to the network estimate in the row. The colors indicate the change in inconsistency when relaxing the consistency assumption for single designs. Diagonal colors show the inconsistency contribution of the corresponding design, whereas off-diagonal colors reflect the change in inconsistency between direct and indirect evidence. Clustering identifies hot spots of inconsistency, helping to locate potential sources for further investigation.

**eFigure 10. Funnel Plot for Pain Intensity**

**

**

**eFigure 11. Network Splitting Analysis for Pain Intensity**

**
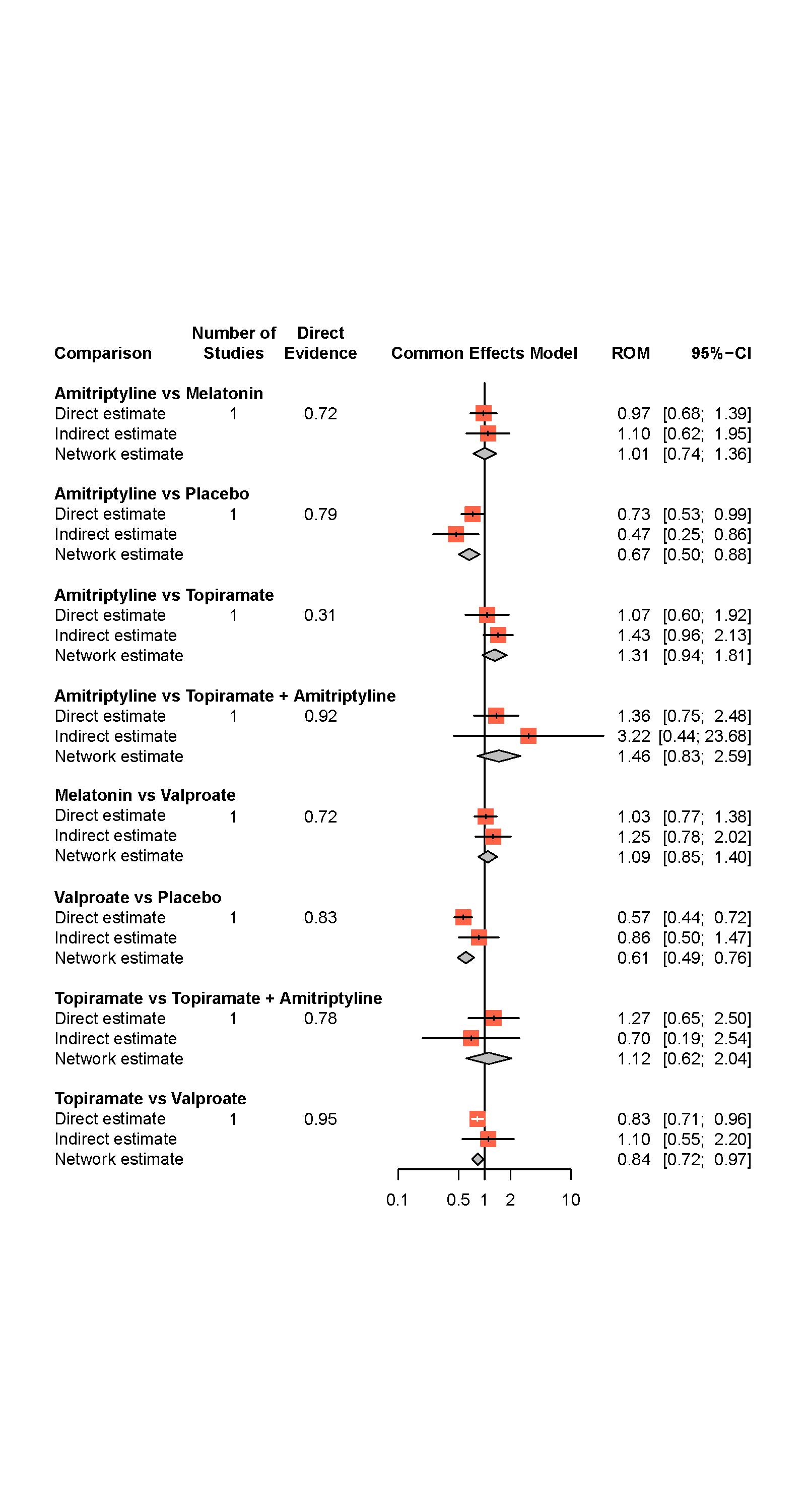
**

This figure presents the network splitting analysis for pain intensity, which examines the consistency between direct and indirect evidence within the network meta-analysis. By splitting the network into these two types of evidence, the analysis evaluates whether the conclusions drawn from the meta-analysis are consistent regardless of the source of evidence, ensuring the robustness and reliability of the overall results.

**
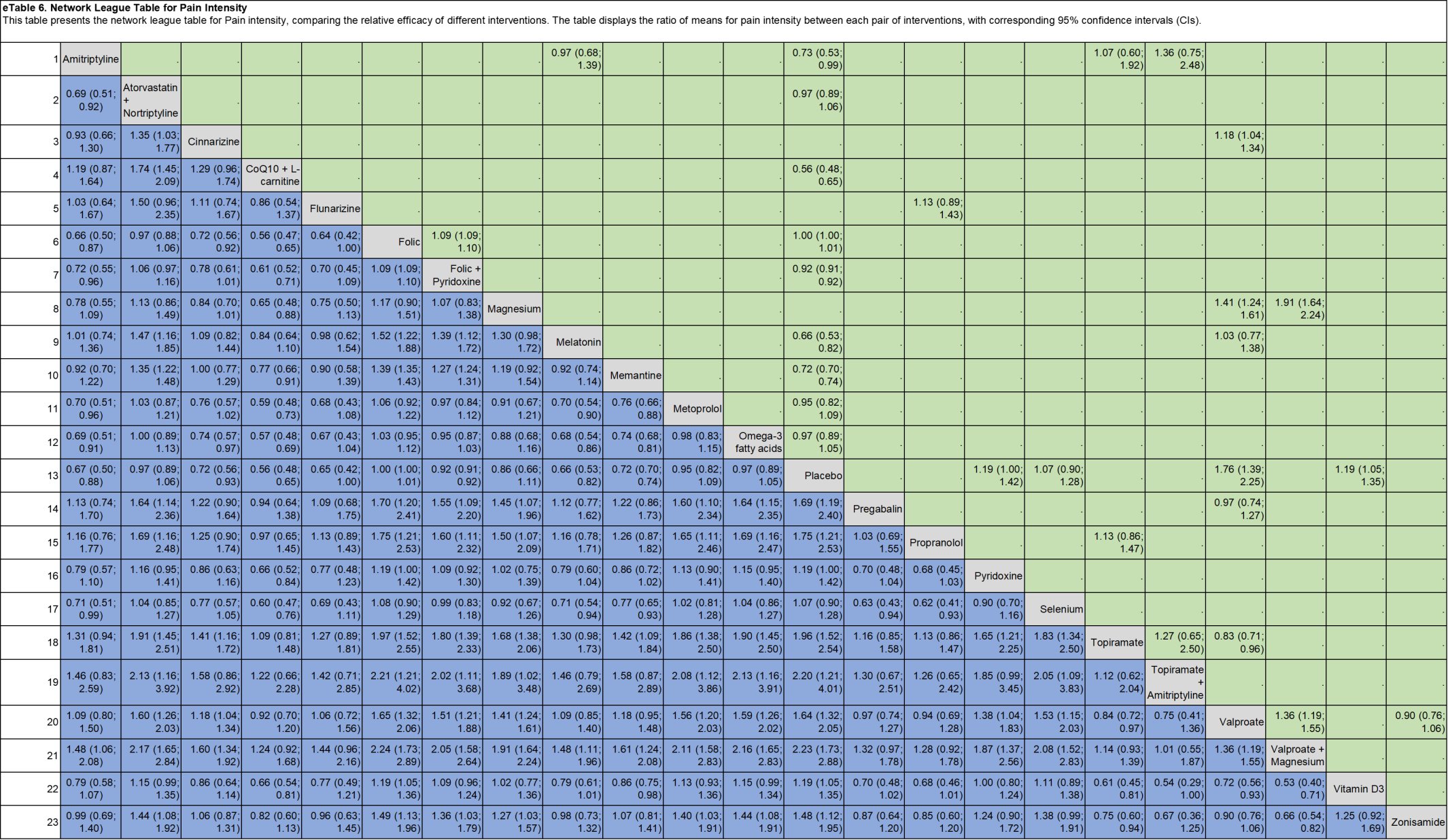
**

**eFigure 12. Network Meta-Analysis Heatmap for Pain Intensity**

**
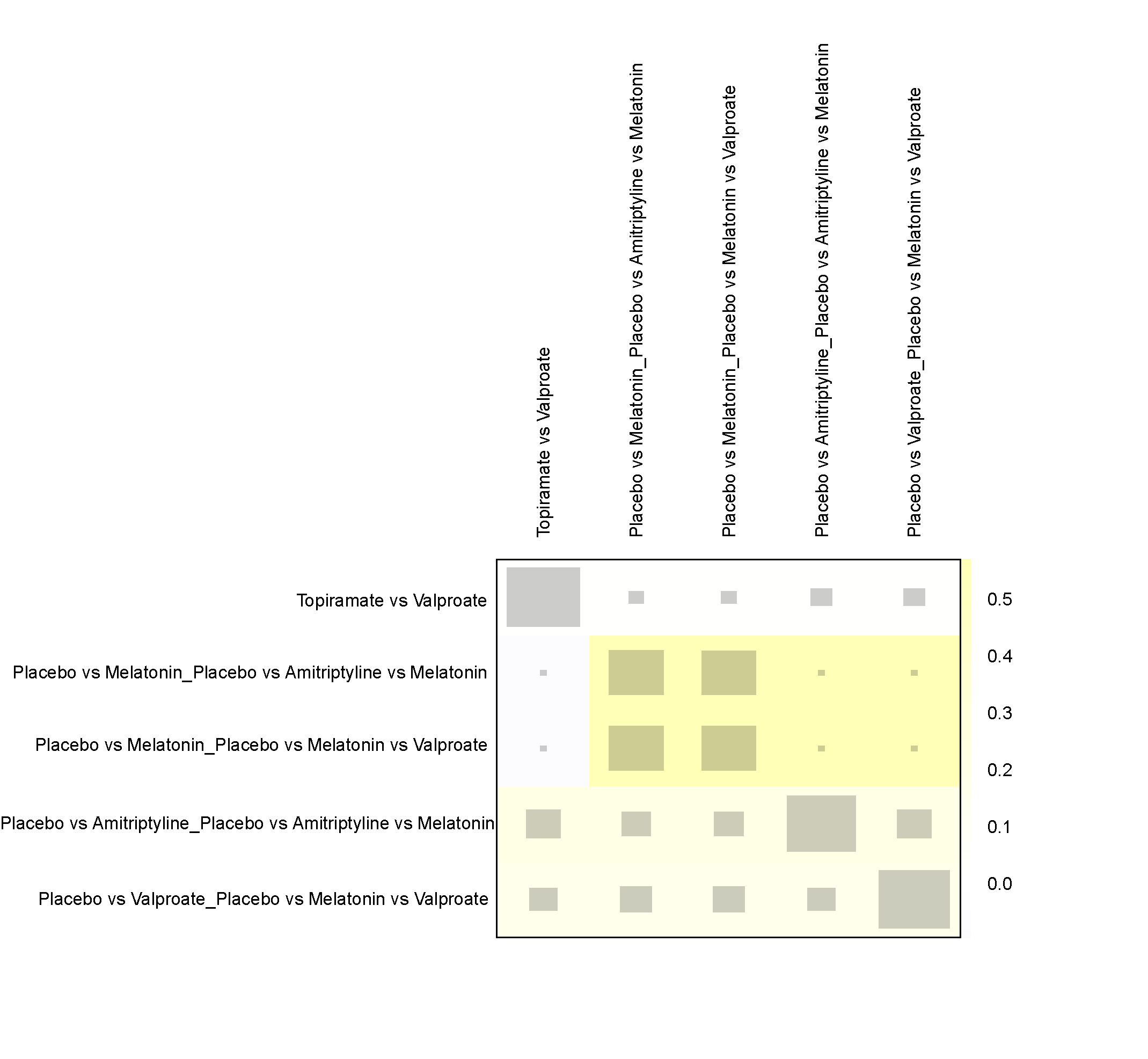
**

The net heat plot is a matrix visualization that highlights areas of inconsistency within the network-meta analysis. Each gray square's area represents the contribution of the direct estimate from the design in the column to the network estimate in the row. The colors indicate the change in inconsistency when relaxing the consistency assumption for single designs. Diagonal colors show the inconsistency contribution of the corresponding design, whereas off-diagonal colors reflect the change in inconsistency between direct and indirect evidence. Clustering identifies hot spots of inconsistency, helping to locate potential sources for further investigation. Designs involving three or more treatments are marked with an underscore following the treatments of the design.

**eFigure 13. Funnel Plot for Migraine Duration**

**

**

**eFigure 14. Network Splitting Analysis for Migraine Duration**

**
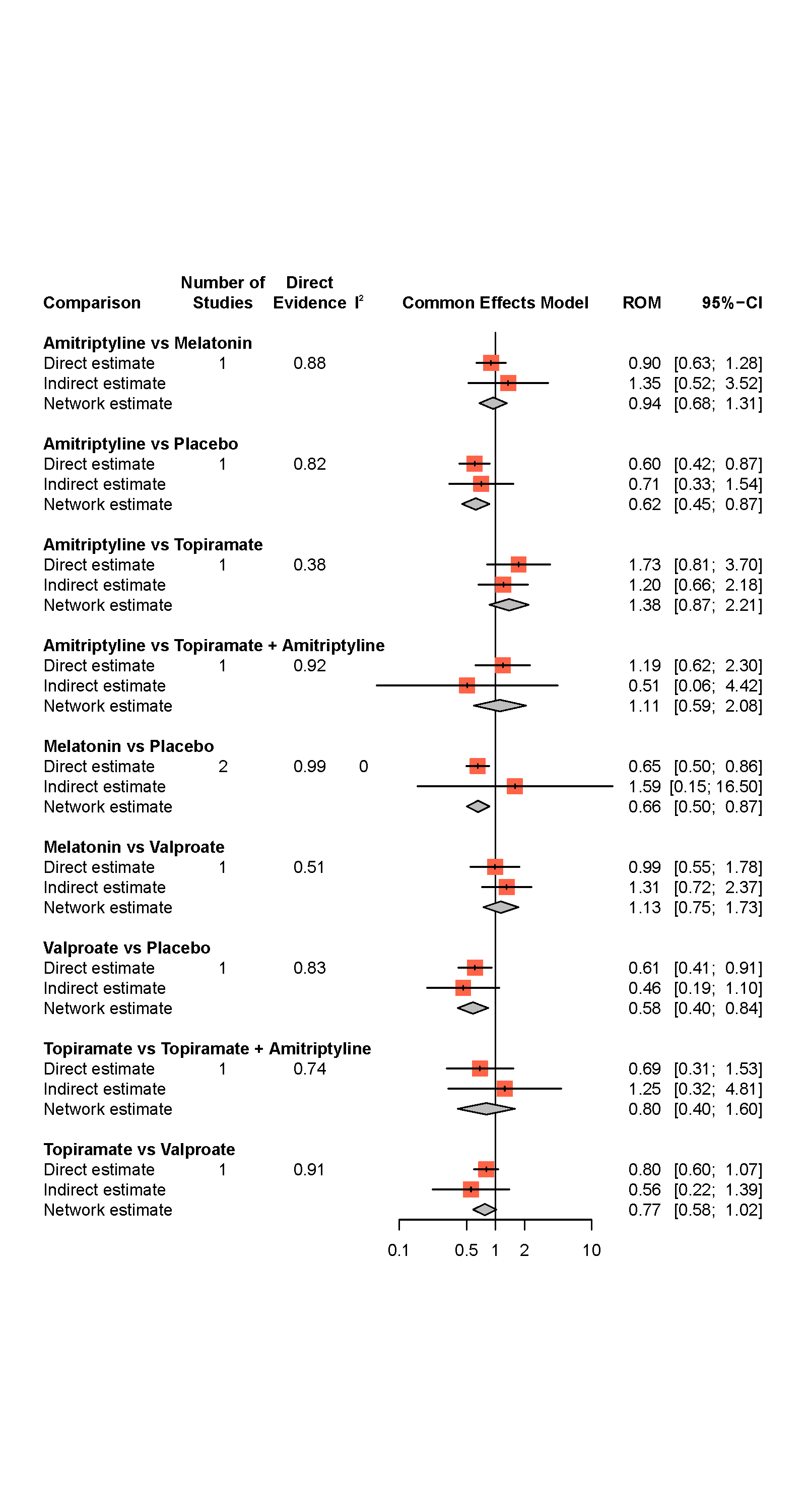
**

This figure presents the network splitting analysis for migraine duration, which examines the consistency between direct and indirect evidence within the network meta-analysis. By splitting the network into these two types of evidence, the analysis evaluates whether the conclusions drawn from the meta-analysis are consistent regardless of the source of evidence, ensuring the robustness and reliability of the overall results.

**
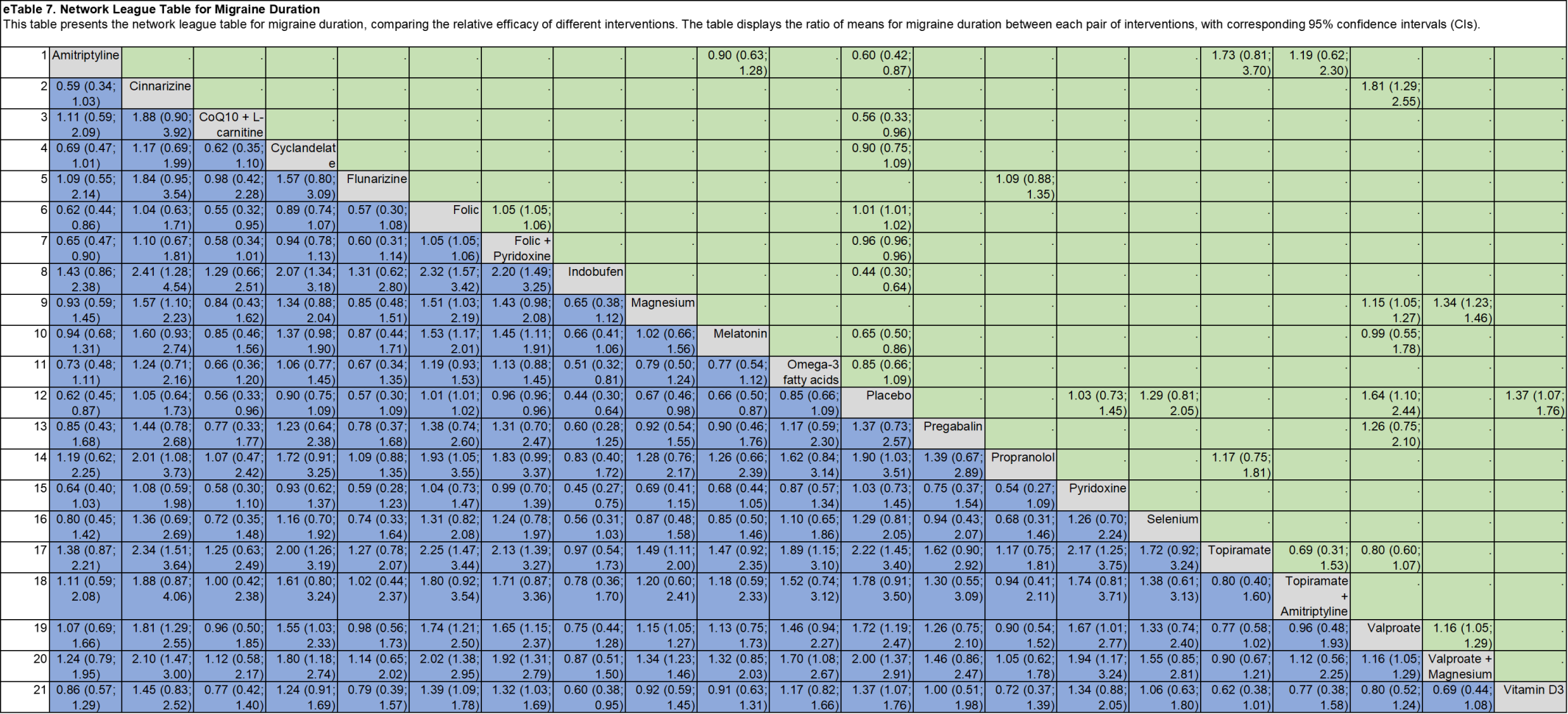
**

**eFigure 15. Network Meta-Analysis Heatmap for Migraine Duration**

**
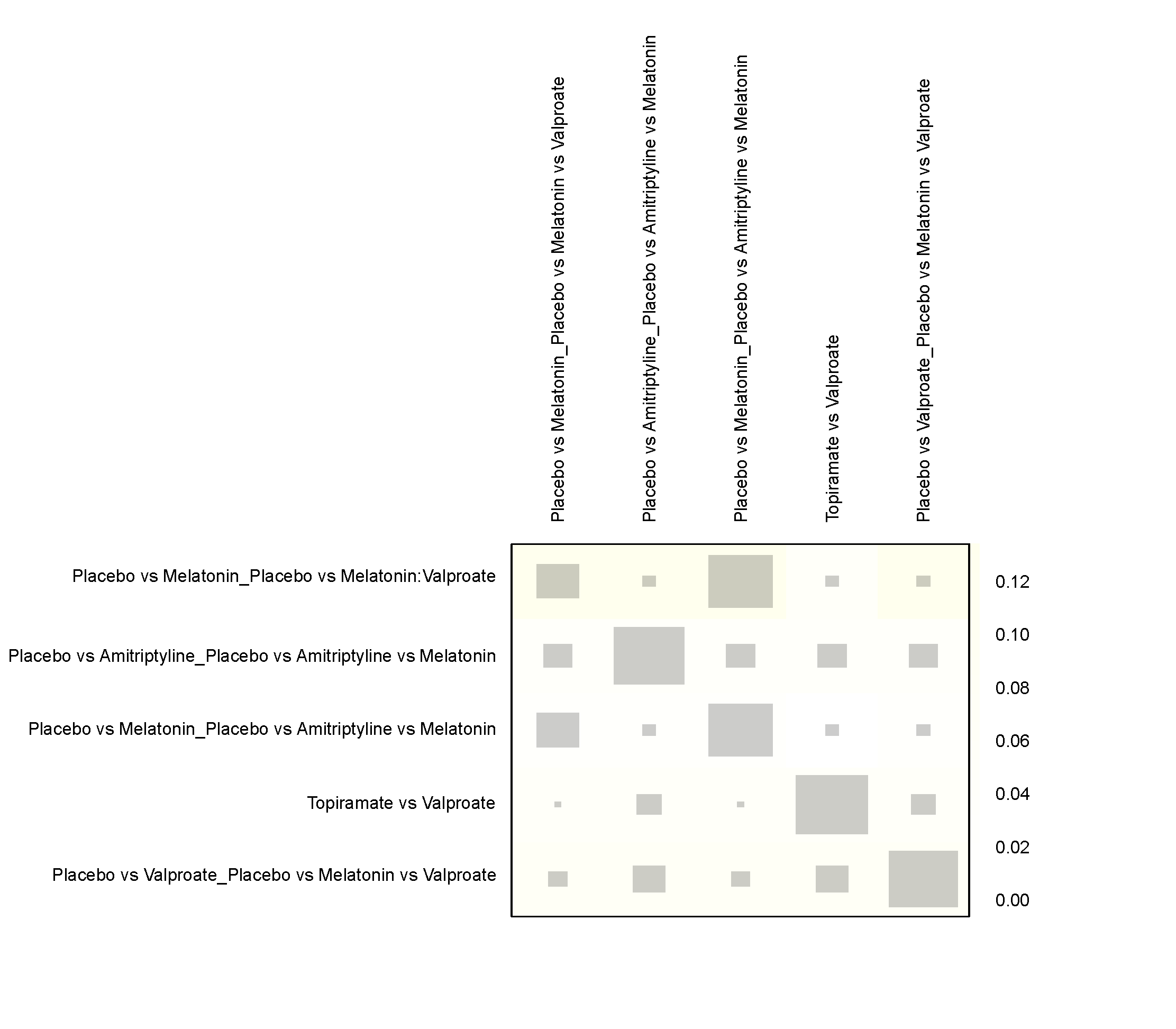
**

The net heat plot is a matrix visualization that highlights areas of inconsistency within the network-meta analysis. Each gray square's area represents the contribution of the direct estimate from the design in the column to the network estimate in the row. The colors indicate the change in inconsistency when relaxing the consistency assumption for single designs. Diagonal colors show the inconsistency contribution of the corresponding design, whereas off-diagonal colors reflect the change in inconsistency between direct and indirect evidence. Clustering identifies hot spots of inconsistency, helping to locate potential sources for further investigation. Designs involving three or more treatments are marked with an underscore following the treatments of the design.

**eFigure 16. Funnel Plot for Quality-of-Life Outcome**

**

**


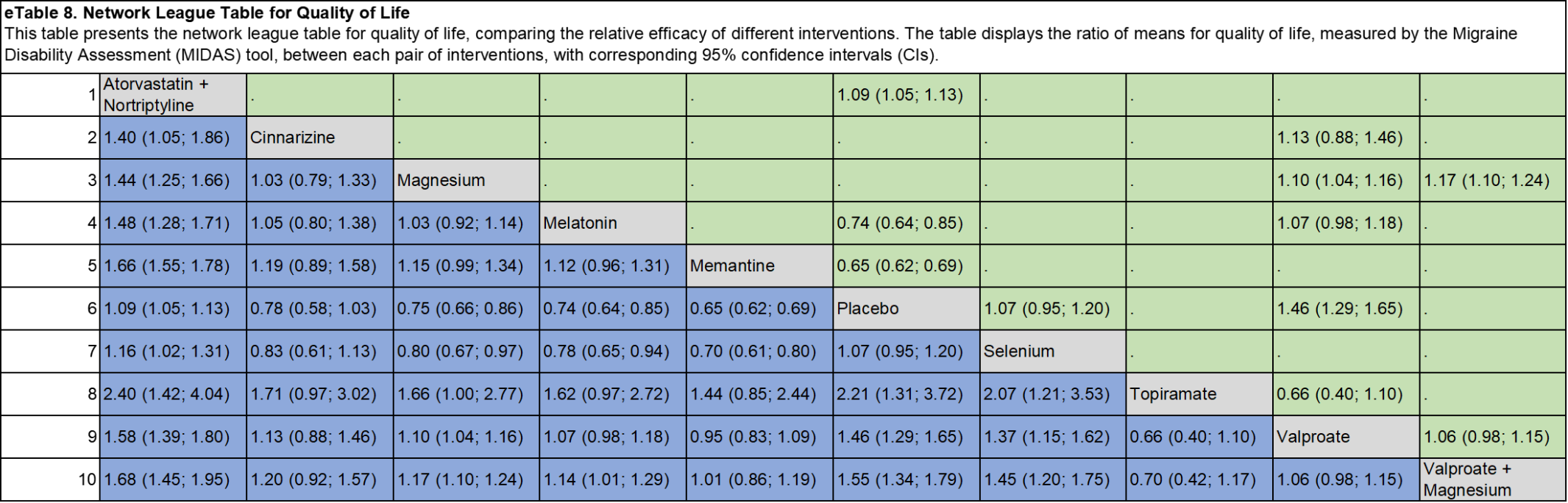


**eFigure 17. Forest Plot of Adverse Events of Each Intervention Compared With Placebo**

**
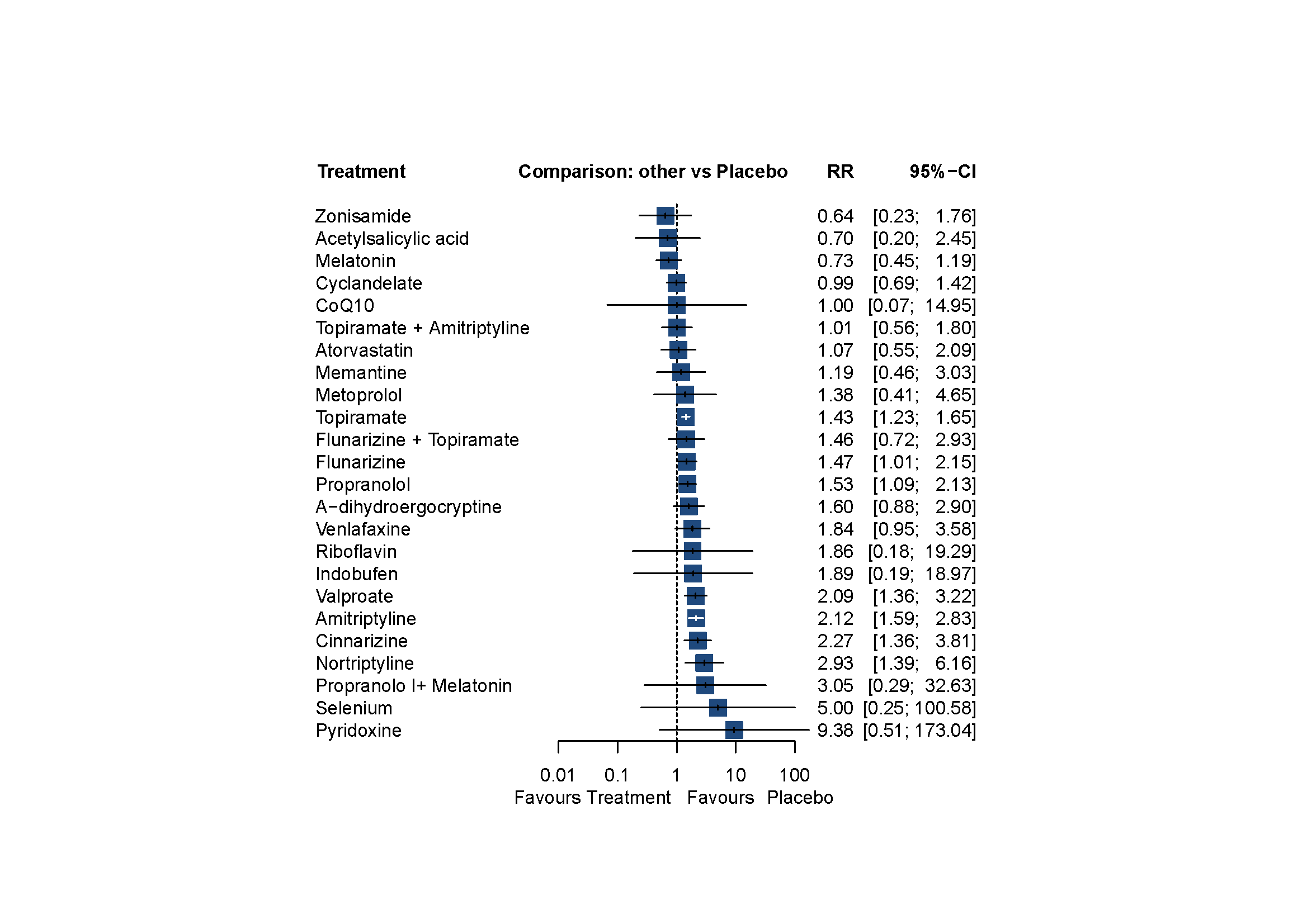
**

**eFigure 18. Network Graph for Adverse Events**

**
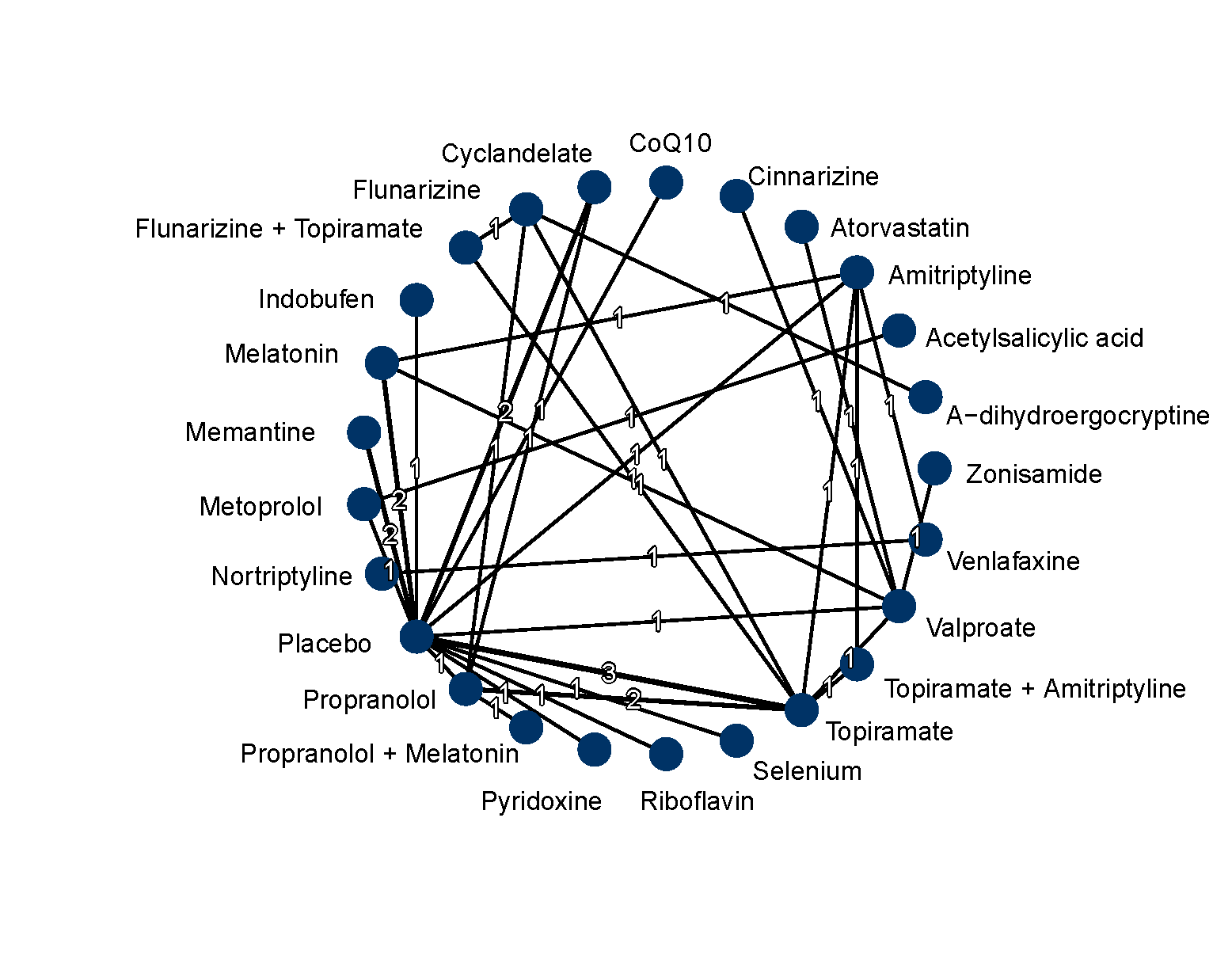
**

**eFigure 19. Funnel Plot for Safety**





**
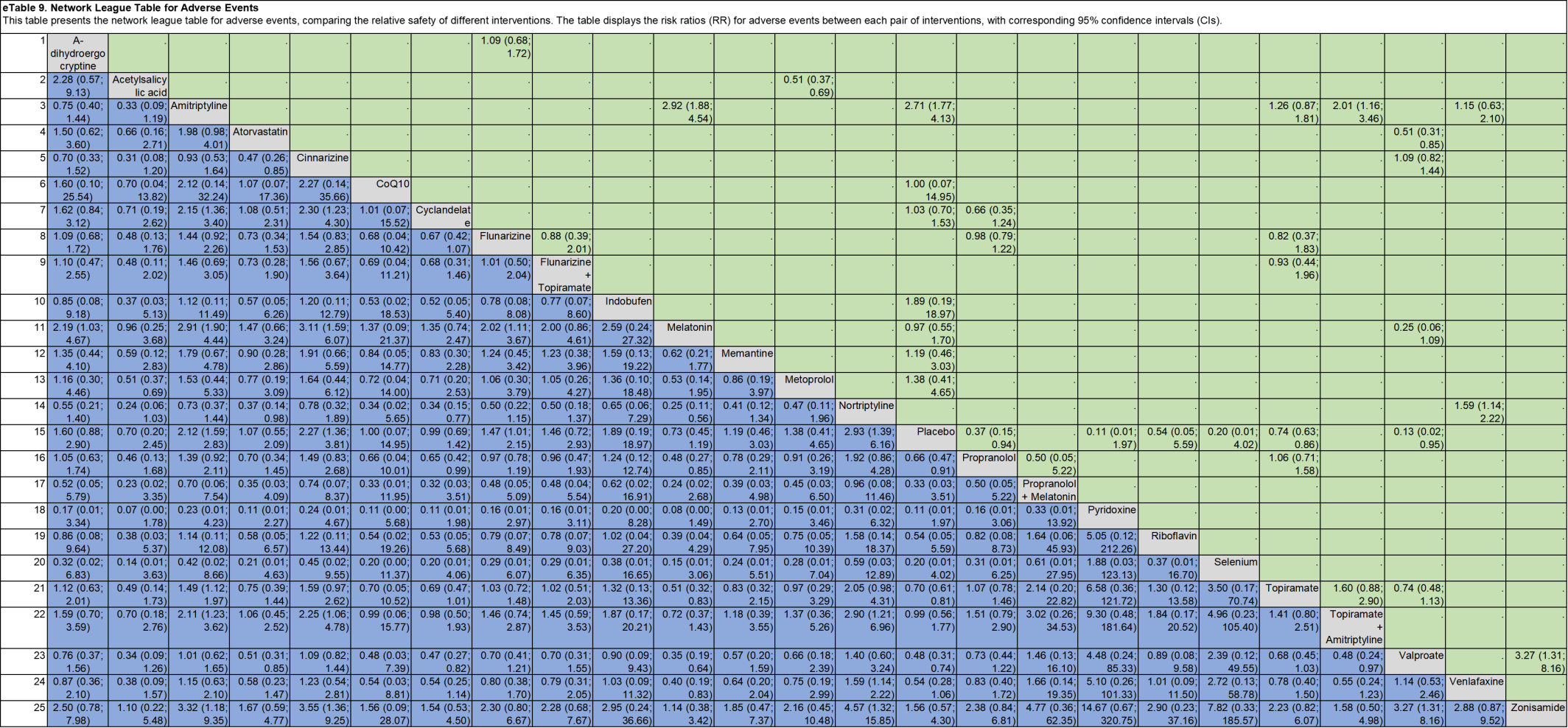
**

**eFigure 20. Net Splitting Analysis for Adverse Events**

**
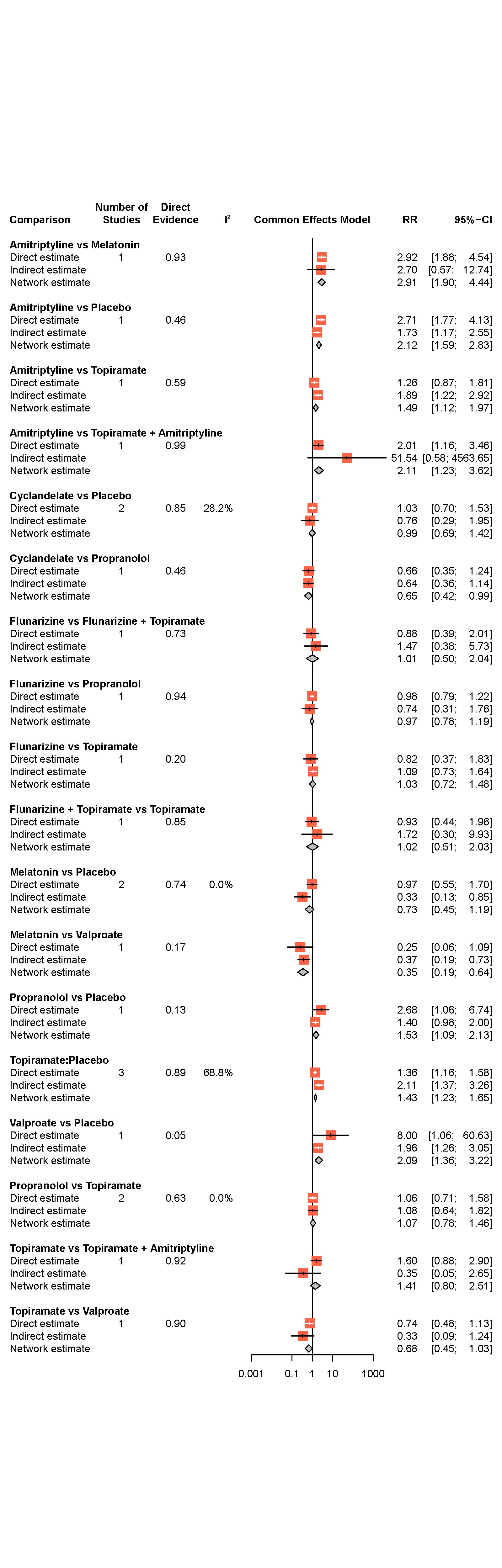
**

This figure presents the network

splitting analysis for adverse events,

which examines the consistency

between direct and indirect evidence

within the network meta-analysis. By

splitting the network into these two

types of evidence, the analysis

evaluates whether the conclusions

drawn from the meta-analysis are

consistent regardless of the source of

evidence, ensuring the robustness and

reliability of the overall results.

**eFigure 21. Network Meta-Analysis Heatmap for Adverse Events**

**
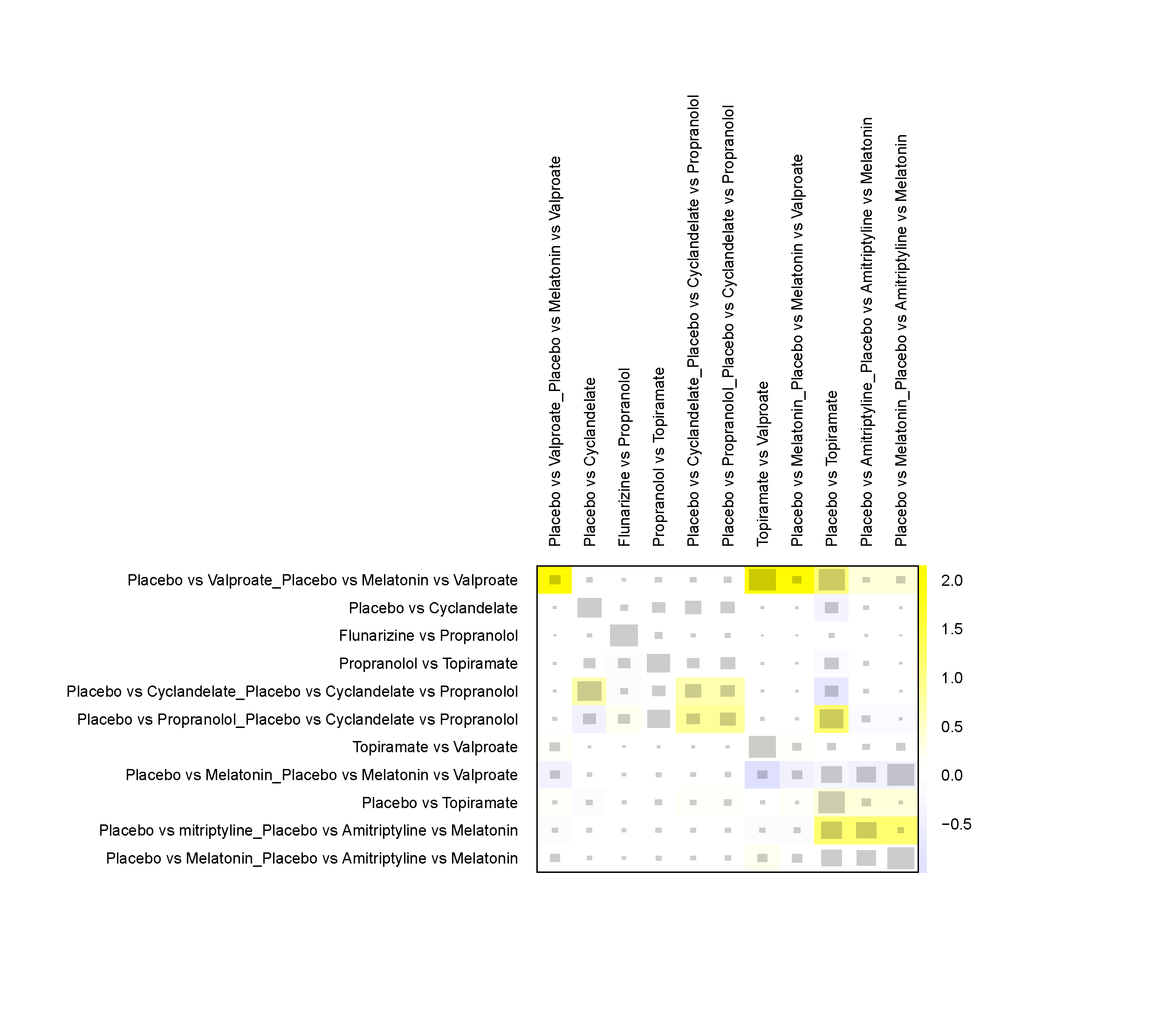
**

The net heat plot is a matrix visualization that highlights areas of inconsistency within the network meta-analysis. Each gray square's area represents the contribution of the direct estimate from the design in the column to the network estimate in the row. The colors indicate the change in inconsistency when relaxing the consistency assumption for single designs: cool colors (e.g., blue) indicate an increase in inconsistency, while warm colors (e.g., red) indicate a decrease. Diagonal colors show the inconsistency contribution of the corresponding design, whereas off-diagonal colors reflect the change in inconsistency between direct and indirect evidence. Clustering identifies hot spots of inconsistency, helping to locate potential sources for further investigation. Designs involving three or more treatments are marked with an underscore following the treatments of the design.
